# Research on mammary gland hyperplasia compound patents’ herbal combination rules based on complex system entropy clustering: A review

**DOI:** 10.1097/MD.0000000000042549

**Published:** 2025-06-20

**Authors:** Xujie Yang, Xiaohua Pei, Hong Zhang, Wanyue Zhang

**Affiliations:** a Department of Medical History Literature, Hebei University of Chinese Medicine, Hebei, China; b Xiamen Hospital, Beijing University of Chinese Medicine, Traditional Chinese Medicine Surgery, Fujian, China.

**Keywords:** complex system, entropy cluster, herbal compound, mammary gland hyperplasia, prescription rule

## Abstract

The aim of this study was to investigate the combination rules of herbal compound patents for the treatment of mammary gland hyperplasia (MGH). Herbal compound patents (oral drugs) for the treatment of MGH from the past 31 years were collected, and a database was created. Complex system entropy clustering was used to analyze the prescription rule of herbal compound patents for the treatment of MGH. In total, 765 MGH herbal compound authorized patents, including 136 Chinese herbs, were identified. High-frequency herbs, herbal pairs, and core associations were obtained by complex system entropy clustering. MGH herbal compound patents are known to activate blood circulation, remove stasis, soothe the liver, regulate *Qi*, clear heat, detoxify, reduce lumps, soften hard mass, dissolve knots, eliminate phlegm, resolve stagnation, expel pus, tonify *Qi* and blood, nourish *Yin*-essence, and generate muscle. A combination of 2 or more effective herbs can achieve stronger therapeutic effects (such as activate blood circulation + activate *Qi* circulation, supplement blood + promote blood circulation, nourish blood + promote *Qi* circulation, soothe the liver + promote blood circulation, soothe the liver + promote *Qi* circulation, soothe the liver + soften lumps and dissipate nodules, reduce swelling/dissipate nodules + promote *Qi* circulation, promote *Qi* circulation + reduce swelling/discharge pus, promote *Qi* circulation + tonify spleen/promote water metabolism/clear damp, reduce swelling/disperse nodules/discharge pus + activate blood circulation, etc). The correlation of MGH with herbal compound patents can be effectively analyzed by complex system entropy clustering.

## 1. Introduction

Mammary gland hyperplasia (MGH) is a common gynecological disease among young and middle-aged women whose incidence is increasing year by year, which seriously affects the patient’s quality of life. Androgens, progesterone (P), tamoxifen, bromocriptine, danazol and other hormonal drugs are commonly used to treat this disease. However, although these drugs can improve the symptoms of MGH, their side effects are severe. Notably, the traditional Chinese medicine (TCM) compound prescription for treating MGH shows its effectiveness in the treatment of this disease. In long-term clinical practice, TCM has accumulated numerous herbal compounds with outstanding curative effects.

The herbal combination pattern determined by complex system-based entropy clustering could enhance the accuracy of clinical prescriptions, making the selection of herbal core combinations easier and more accurate.^[1]^

There are many academic cores in the MGH herbal compound patents, which will have remarkable curative effect on clinical treatment. In this study, the patents for oral herb prescriptions for MGH treatment from the past 30 years were selected for analysis. Herbal frequency, association, and composition compatibility regularity were investigated to guide the clinical prescription and development of new drugs.

**Figure 1.**
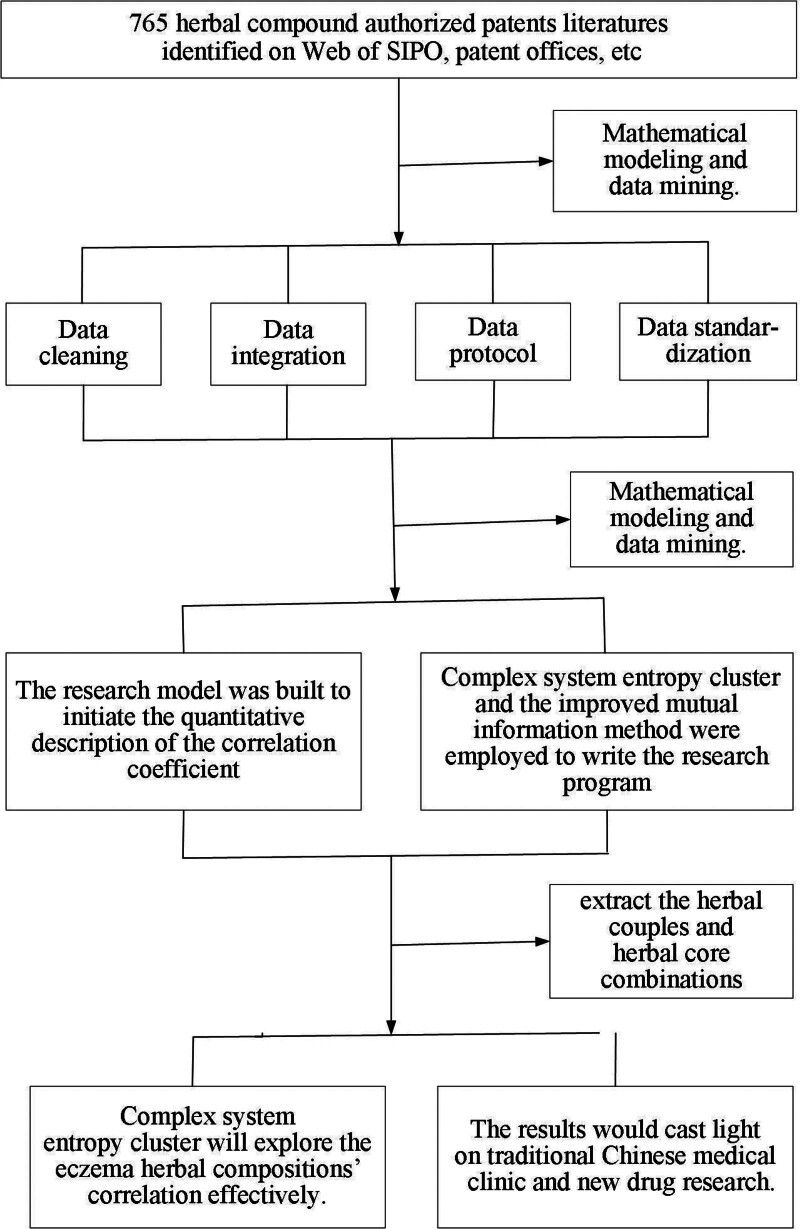
Algorithm of the research process.

**Figure 2.**
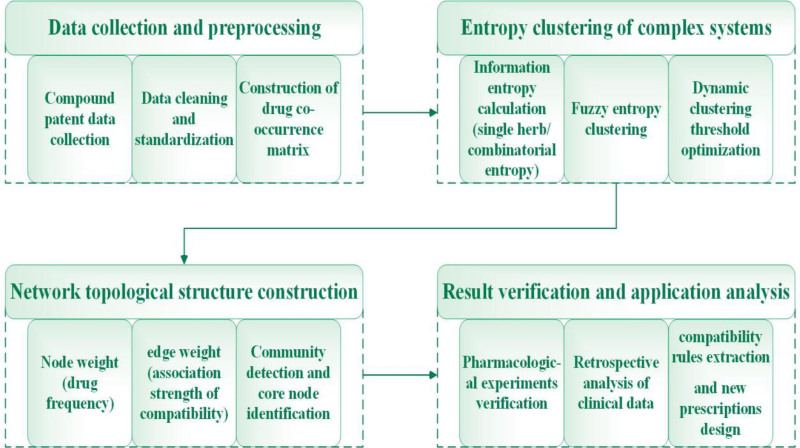
Research process of entropy clustering of complex systems for traditional Chinese medicine compound patents.

**Figure 3.**
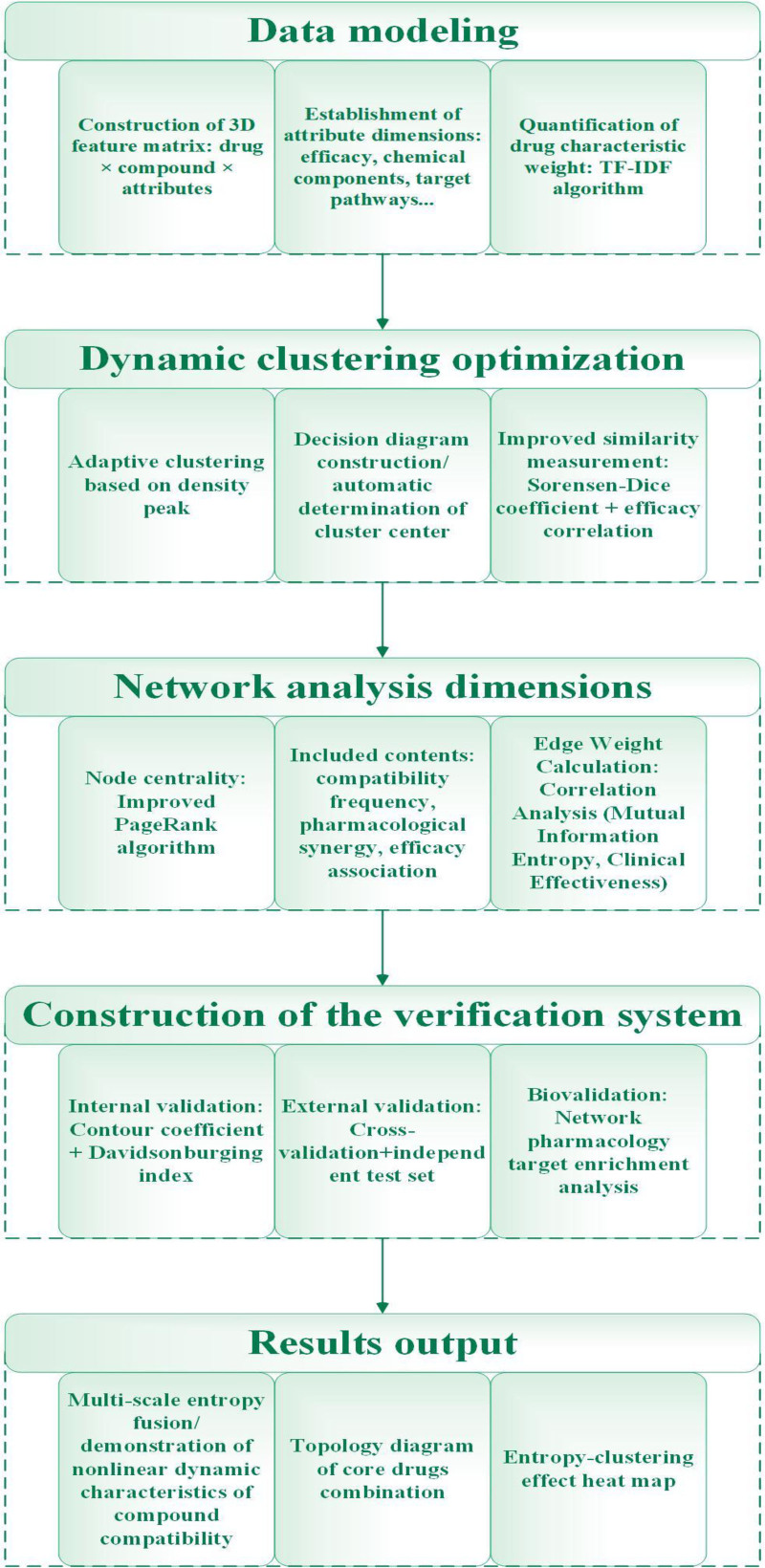
Key steps in entropy clustering research of complex systems.

This study introduces the boundary mutual distance into the mutual information registration measure. By combining mutual information and mutual distance information, the resulting new registration measure improves the mutual information registration measuring function, thus reducing the risk of the conventional maximum mutual information function falling into the extremum and defining the accurate ratio of information about the MGH patent compound composition (Figs. 1–3).

This study aims to investigate the active ingredients, targets, pathways, and mechanisms of drug pairs or drug combinations in the treatment of MGH.

## 2. Materials and methods

### 2.1. Collection and collation of MGH herbal compound patents

The literature search covered the Derwent World Patent Index database, the National Intellectual Property Office patent database, the World Digital Library of Intellectual Property, the Multinational Patent Review Information Search System, TCM enterprises, and related herbal intellectual property organizations. MGH herbal compound authorized patents between January 1993 and December 2023 were retrieved.

### 2.2. Inclusion criteria

(Breast hyperplasia or hyperplasia of mammary glands or cyclomastopathy or lobular hyperplasia of the breast or Benign breast hyperplasia) + (TCM or herbal medicine or natural medicine or herbs) + classification numbers (A61K 36/00 or A61P…). Based on the recall-precision curve, the retrieval formulas were optimized and adjusted dynamically.

### 2.3. Exclusion criteria

1.  Single herb
2.  Integrated use of multiple approaches
3.  Simultaneously treating other diseases
4.  Combined medication

### 2.4. Quality control and validation

All the collected literature on patents for herbal compounds for breast hyperplasia were checked by 3 persons to ensure accuracy and precision.

### 2.5. Database establishment

The EpiData 3.1 system (EpiData Association, Odense, Denmark) was used to develop the data mining software. Each eligible MGH prescription patent herb was assigned a 2-valued variable.^[2]^ When the users click with the mouse on the Chinese herbs involved in a prescription, they automatically assigns them the value “1,” and the remaining unclicked herbs are automatically assigned the value “0.” The database contained 765 patents of compound prescriptions for MGH, including 136 commonly used Chinese herbs.

### 2.6. Mathematical modeling and data mining

Data mining was performed using mathematical entropy modeling and screening out herbal pairs and core combinations with strong efficacy coherence. Complex system entropy clustering and improved mutual information methods were used to construct the research program and create the research model in order to initiate the quantitative description of the correlation coefficient of Chinese medicine. The ultimate purpose was to extract the herbal pairs and herbal core combinations.

Mathematical modeling was used to mine the MGH prescription patents. The most relevant core combinations of Chinese herbs were screened out by mathematical calculations, comprising core herbal pairs and herbal teams. The improved mutual information methods, complex system entropy clustering methods, and unsupervised entropy hierarchical clustering methods were used for data analysis. The core formulas of the improved mutual information methods and complex system entropy clustering methods are the following:


$$\begin{array}{l} \;\Delta \mu^{\prime }\left(X_{i},X_{j}\right)=\left\{ \begin{array}{l} \frac{H\left(X_{i}\right)+H\left(X_{j}\right)-H\left(X_{i},X_{j}\right)}{H\left(X_{j}\right)}P_{o}\left(i,j\right)\geq \delta ,\;\\ \frac{HX_{i}+HX_{j}-2H\left(X_{i},X_{j}\right)}{H\left(X_{j}\right)}P_{o}\left(i,j\right)<\delta .\; \end{array}\right. \end{array}$$

Unsupervised hierarchical clustering can be described using the following formula: $MI^{\prime }\left(X,Y\right)=\left\{ \begin{array}{l} H\left(X\right)+H\left(Y\right)-H\left(X\cup Y\right)\;pro\left(X,Y\right)=\;0,\;\\ H\left(X\right)+H\left(Y\right)-2*H\left(X\cup Y\right)pro\left(X,Y\right)>0,\; \end{array}\right.$

where *P*_*o*_(*i*, *j*) denotes the frequency of positive occurrences of the 2 variables *X*_*i*_ and *X*_*j*_; δ represents the threshold value, selecting the appropriate threshold can separate the positive correlation from the negative correlation and avoid the interference caused by incorrect data; *H*(*X*) and *H*(*Y*) denote the information entropy of *X* and *Y*, respectively, whereas *H*(*X* ∪ *Y*) denotes joint entropy.

The above process can also be expressed as follows:


$$\begin{array}{l} \mu \left(X_{i},X_{j}\right)=\;C\left(X_{i}\right)+C\left(X_{j}\right)\;C\left(X_{i},X_{j}\right)\;\\ \;\;\;\;\;\;\;\;\;\;\;\;\;\;\;\;\;\;\;=\underset{i}{\sum }\underset{j}{\sum }q\left(X_{i},\;X_{j}\right)\;\;\;lg\frac{q\left(X_{i},\;\;X_{j}\right)}{q\left(X_{i}\right)q\left(X_{j}\right)}, \end{array}$$ 1

$$\begin{array}{l} \mu_{ij}=\frac{u\left(X_{i},\;\;\;X_{j}\right)}{\sqrt{C\left(X_{i}\right)}\sqrt{C\left(X_{j}\right)}}, \end{array}$$ 2

$$\begin{array}{l} \mu_{\left(X_{i},\;\;\;X_{j}\right)}=\left\{ \begin{array}{l} C\left(X_{i}\right)+C\;\left(X_{j}\right)-C\;\left(X_{i},\;\;X_{j}\right)\;\;Q_{o}\left(i,j\right)\geq \delta ,\;\\ C\left(X_{i}\right)+C\left(X_{j}\right)-bC\left(X_{i},\;\;X_{j}\right)Q_{o}\left(i,j\right)<\delta ,\; \end{array}\right. \end{array}$$ 3

$$\begin{array}{l} \Delta \mu \left(X_{i},X_{j}\right)=\left\{ \begin{array}{l} \frac{C\left(X_{i}\right)+C\left(X_{j}\right)-C\left(X_{i},\;X_{j}\right)}{C\left(X_{j}\right)}Q_{o}\left(i,\;j\right)\geq \delta ,\;\\ \frac{C\left(X_{i}\right)+C\left(X_{j}\right)-C\left(X_{i},\;XX_{j}\right)}{C\left(X_{j}\right)}Q_{o}\left(i,j\right)<\delta ,\; \end{array}\right. \end{array}$$ 4

where *q*(*X*_*i*_) and *q*(*X*_*j*_) represent the probability density function of variable *X*_*i*_; variables *X*_*j*_, *C*(*X*_*i*_), and *C*(*X*_*j*_) represent the entropy of variable *X*_*i*_ and variate *X*_*j*_ respectively; *C*(*X*_*i*_, *X*_*j*_) denotes the combination entropy of variable *X*_*i*_ and variate *X*_*j*_; *u*_*ij*_ is a dimensionless quantity. When variable *X*_*i*_ and variable *X*_*j*_ are in perfect correlation, the value of variable *u*_*ij*_ is 1. When variable *X*_*i*_ and variate *X*_*j*_ are completely independent, the value of variable *u*_*ij*_ is 0. Generally, the value of variable *u*_*ij*_ is between 0 and 1, whereas variable *u*_*ij*_ is equal to variable *u*_*ji*_. *Q*_*o*_(*i*, *j*) represents the positive frequency of variable *X*_*i*_ and variable *X*_*j*_, *b* is a penalty coefficient, and δ is the threshold value.

## 3. Results

**Figure 4.**
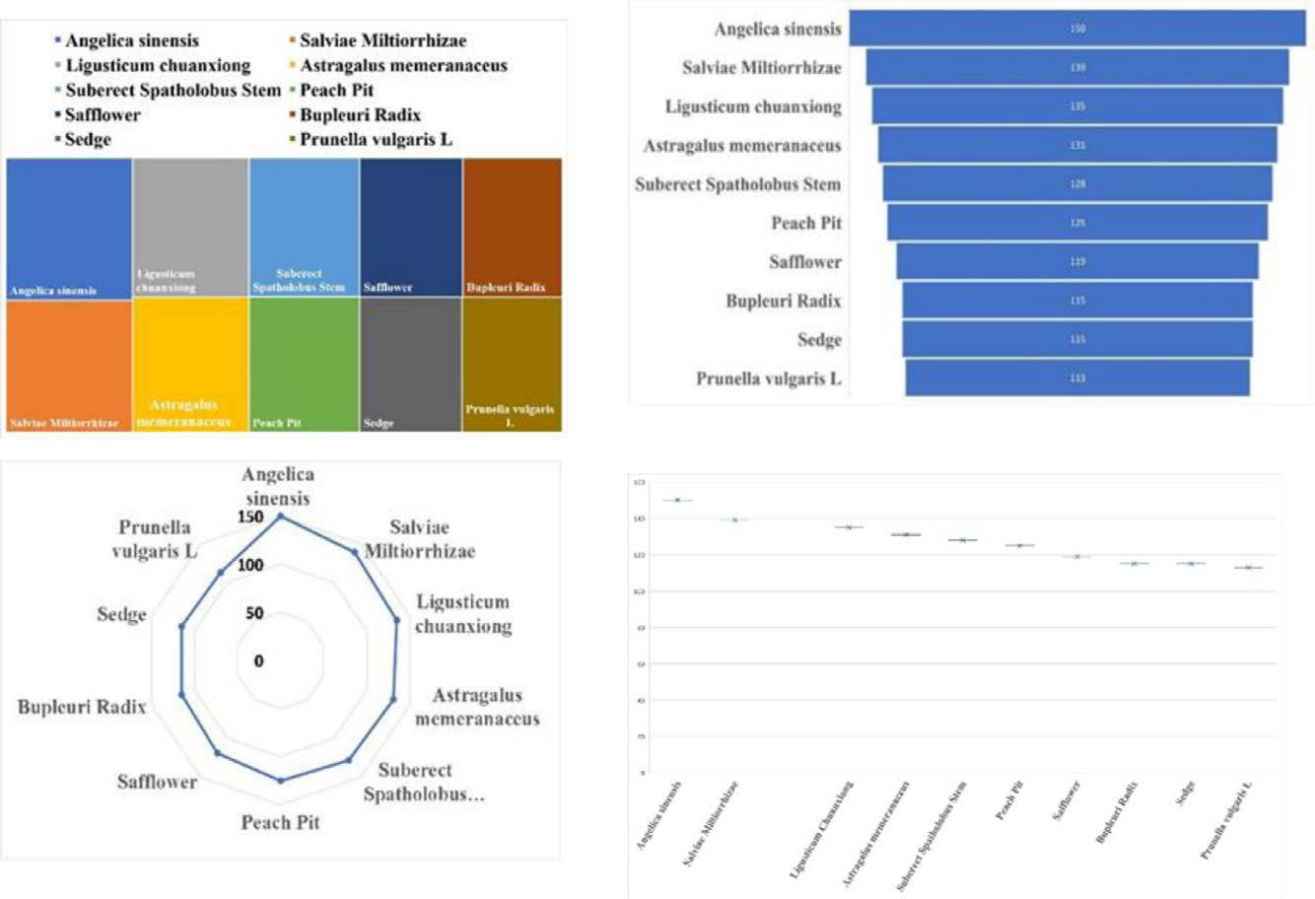
Frequency ratios of various functions’ herbs.

**Figure 5.**
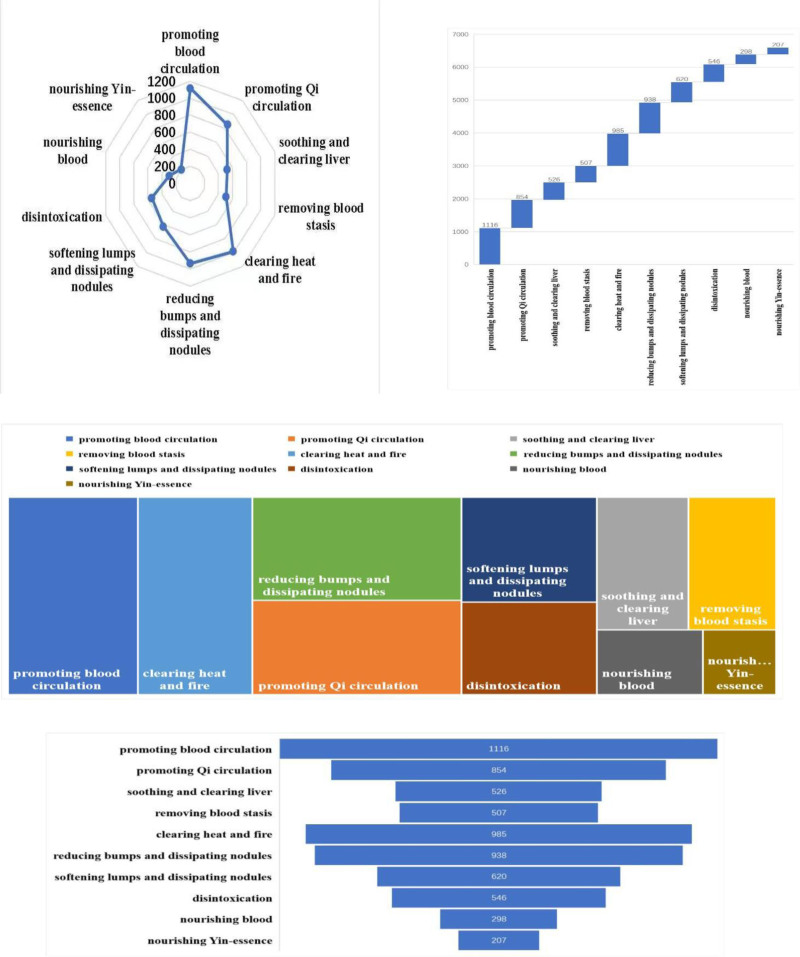
Herbal core combinations complex network.

**Figure 6.**
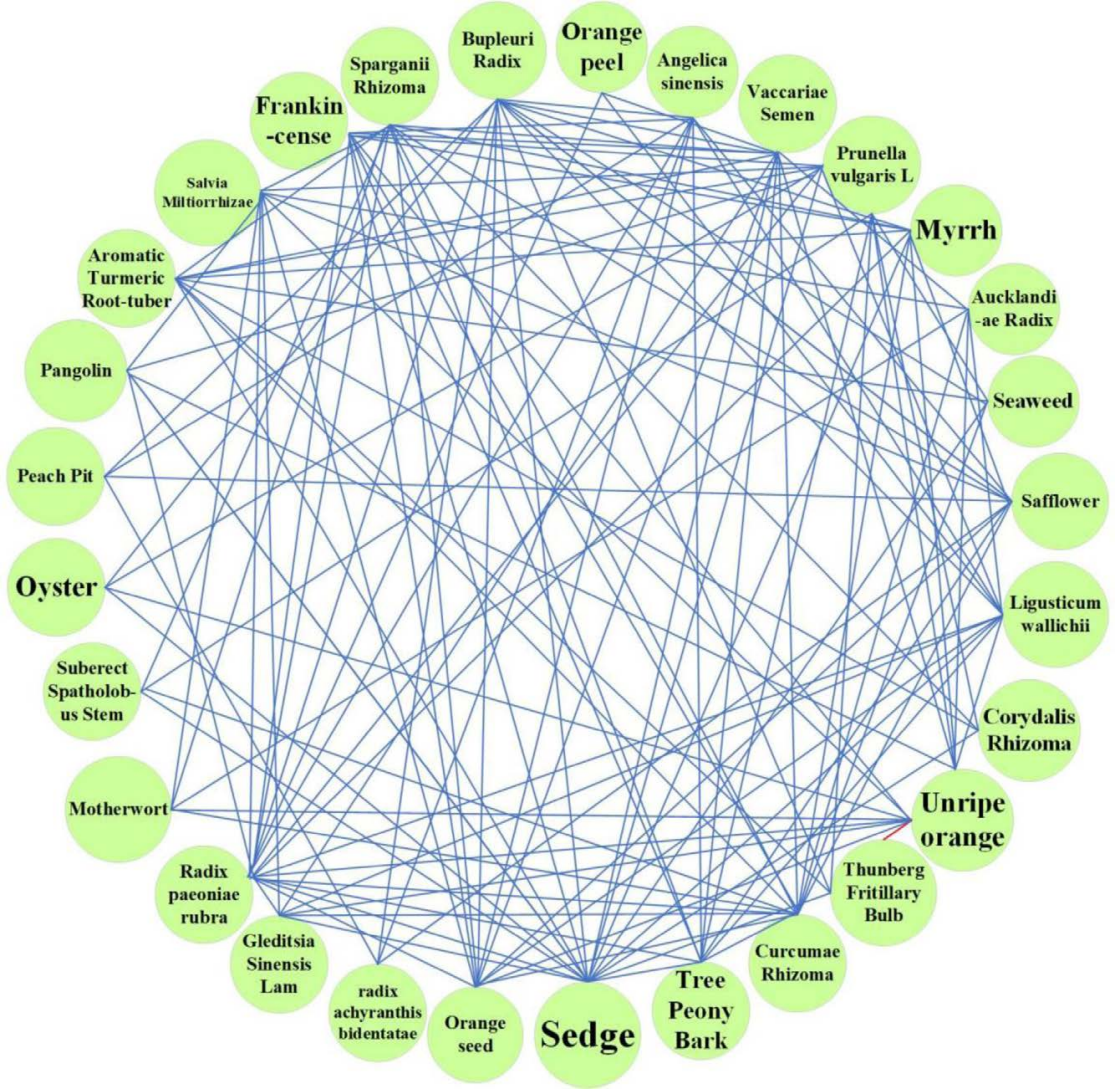
Drug pairs association.

**Figure 7.**
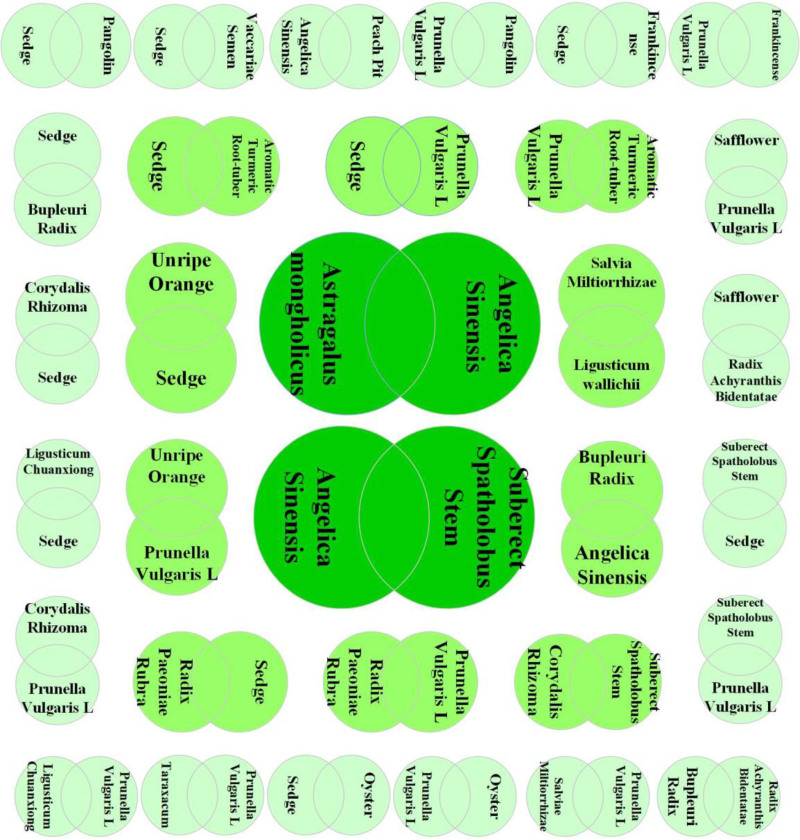
Frequency ratios of high frequency herbs.

**Figure 8.**
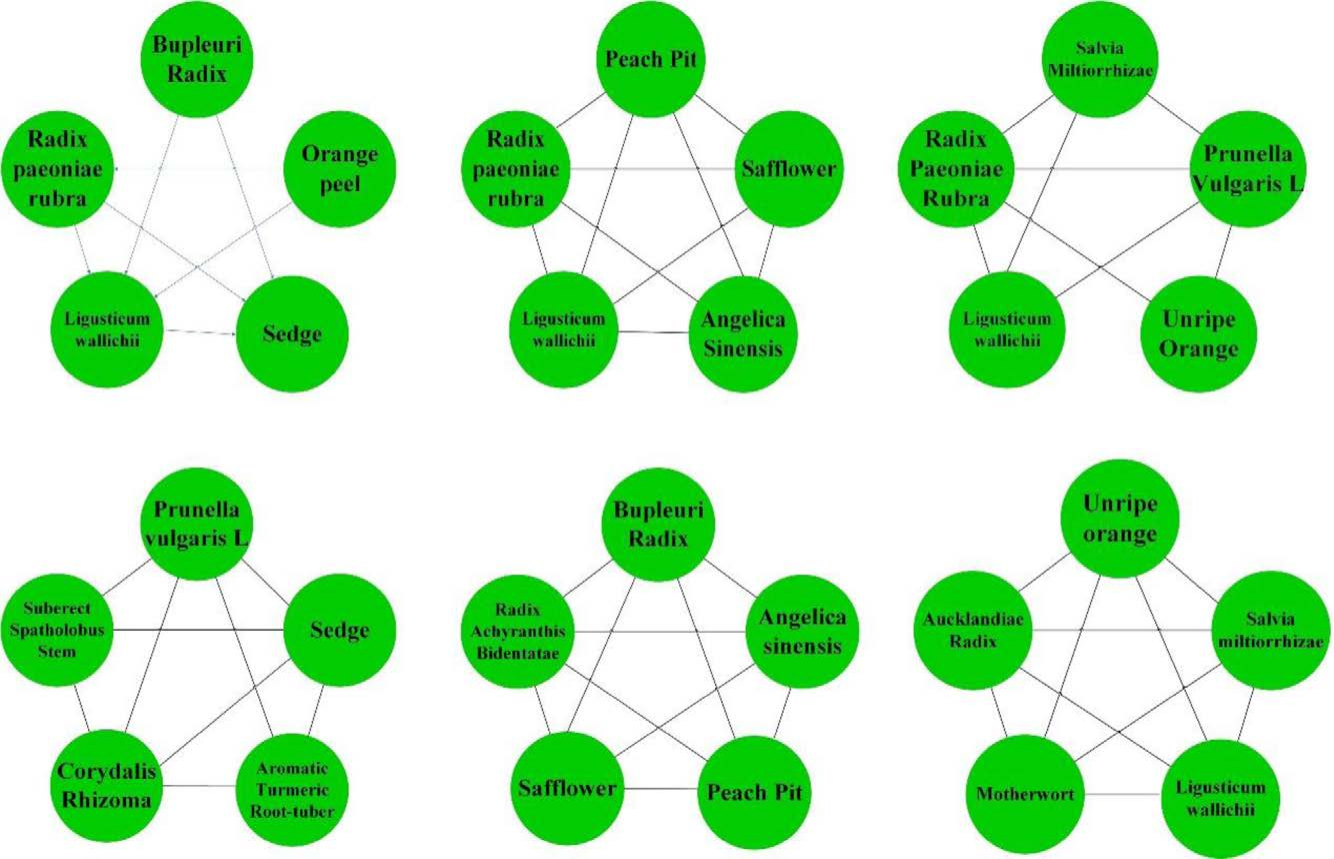
Herbal group complexation.

1. In this study, a total of 765 prescriptions in the database were analyzed and screened using the methods mentioned above. A total of 136 Chinese herbs with high frequency and 62 regularly used drug pairs were obtained using an improved mutual information method, and 32 groups of core combinations were obtained using complex system-based entropy clustering.
2. The high frequency efficacy, high frequency herbs, herbal pairs, efficacy pairing of Chinese herbs and herbal core combinations are shown in Tables 1–5, respectively.
3. The effects of high frequency herbs are associated with promoting blood circulation, removing blood stasis, detumescing, pain relieving, clearing heat and detoxifying, liver soothing and regulating *Qi*, etc, (Table 1, Fig. 4). The high frequency Chinese herbs include *Angelica sinensis*, *Salvia miltiorrhizae, Ligusticum wallichii*, etc (Table 2, Fig. 5). The herbal pairs consist of 2 herbs that work together synergistically to achieve better performance. The pair consists of 2 herbs with similar efficacy that play a therapeutic role with mutual assistance or 2 herbs with dissimilar efficacy that are superimposed on each other (Tables 3 and 4, Figs. 6 and 7). Herbal core combinations mean that several herbs work together and appear together in most prescriptions (Table 5, Fig. 8).

**Table 1:** High frequency efficacy.

Efficacy	Frequency	Efficacy	Frequency
Promoting blood circulation	1116	Eliminating stagnation	415
Promoting *Qi* circulation	996	Relieving pain	407
Soothing and clearing liver	965	Cooling blood	381
Removing blood stasis	898	Diuresis	376
Clearing heat and fire	867	Discharging pus	353
Reducing bumps and dissipating nodules	832	Dredging menstruation	341
Softening lumps and dissipating nodules	786	Dispelling wind	336
Disintoxication	765	Promote water metabolism	323
Nourishing blood	632	Clearing damp	238
Nourishing *Yin*-essence	543	Diaphoresis	199
Nourishing *Qi*	506	Tonifying spleen	185
Promoting muscle growth	476	Dredging meridians and veins	169
Expelling phlegm	432	Promoting milk excretion	166
Regulating menstruation	425		

**Table 2:** High frequency herbs.

Herb	Frequency	Efficacy	Efficacy classification
*Angelica sinensis*	150	Tonifying blood, promoting blood circulation, regulating menstruation, relieving pain, relieving constipation	Nourishing blood
*Salvia miltiorrhizae*	139	Dispelling blood stasis, relieving pain, promoting blood circulation, clearing heat, regulating menstruation, cooling blood, resolving carbuncle	Promoting blood circulation and removing blood stasis
*Ligusticum wallichii*	135	Regulating *Qi* circulation, promoting blood circulation, expelling wind, relieving pain	Promoting blood circulation and removing blood stasis
*Astragalus mongholicus*	131	Tonifying spleen, nourishing *Qi,* clearing heat, expelling phlegm, detoxifying	nourishing *Qi*
Suberect Spatholobus Stem	128	Activating blood circulation, nourishing blood, regulating menstruation, relieving pain, relaxing tendons, dredging channels and collaterals	Promoting blood circulation and removing blood stasis
Peach Pit	125	Promoting blood circulation, dispelling blood stasis, defecating, relieving cough, relieving asthma	Promoting blood circulation and removing blood stasis
Safflower	119	Promoting blood circulation, dredging channels and collaterals, dispersing stasis, relieving pain	Promoting blood circulation and removing blood stasis
Bupleuri Radix	115	Relieving external symptoms, clearing heat, soothing liver, relieving depression, elevating *Yang*-essence, reducing fever, intercepting malaria	Soothing liver
Sedge	115	Regulating *Qi* circulation, relieving depression, regulating menstruation, relieving pain	Regulating *Qi* circulation
*Prunella vulgaris* L	113	Clearing liver heat, clearing fire, brightening eyes, dispersing knots, reducing swelling	Softening lumps and dissipating nodules
Aromatic Turmeric Root-tuber	113	Promoting blood circulation, relieving pain, regulating *Qi* circulation, relieving depression, cooling blood, clearing heart	Promoting blood circulation and removing blood stasis
*Gleditsia sinensis* Lam	112	Reducing swelling, detoxifying, discharging pus	Clearing phlegm
*Thunberg fritillary* bulb	111	Clearing heat, dissipating nodules, expelling phlegm, relieving cough	Clearing phlegm
Curcumae Rhizoma	108	Regulating *Qi* circulation, promoting blood circulation, eliminating accumulation, relieving pain	Promoting blood circulation and removing blood stasis
Radix paeoniae rubra	108	Clearing heat, ooling blood, promoting blood circulation, dispelling blood stasis, relieving pain	Clearing heat and cooling blood
Unripe orange	108	Soothing liver, regulating *Qi* circulation, eliminating accumulation, eliminating stagnation	Regulating *Qi* circulation
Corydalis Rhizoma	105	Promoting blood circulation, regulating *Qi* circulation, relieving pain	Promoting blood circulation and removing blood stasis
Motherwort	103	Promoting blood circulation, regulating menstruation, diuresis, reducing swelling, clearing heat, detoxifying	Promoting blood circulation and removing blood stasis
Vaccariae Semen	101	Promoting blood circulation, regulating menstruation, promoting milk excretion, reducing swelling, diuresis	Promoting blood circulation and removing blood stasis
Aucklandiae Radix	101	Regulating *Qi* circulation, relieving pain, tonifying, spleen, helping digestion	Regulating *Qi* circulation
Orange peel	98	Regulating *Qi* circulation, tonifying spleen, clearing damp, expelling phlegm	Regulating *Qi* circulation
Pangolin	98	Regulating menstruation, promoting milk excretion, reducing swellin, discharging pus, expelling wind, dredging channels and collaterals	Promoting blood circulation and removing blood stasis
Frankincense	98	Promoting blood circulation, relieving pain, reducing swelling, promoting muscle growth	Promoting blood circulation and removing blood stasis
Sparganii Rhizoma	97	Promoting blood circulation, regulating *Qi* circulation, eliminating accumulation, relieving pain	Promoting blood circulation and removing blood stasis
Myrrh	97	Dispelling blood stasis, relieving pain, reducing swelling, promoting muscle growth	Promoting blood circulation and removing blood stasis
Tree Peony Bark	97	Clearing heat, cooling blood, promoting blood circulation, dispelling blood stasis	Promoting blood circulation and removing blood stasis
Chinaberry fruit	97	Soothing liver, clearing heat, regulating *Qi* circulation, relieving pain	Regulating *Qi* circulation
Oyster	95	Calming nerves, nourishing *Yin*-essence, softening lumps, dissipating nodules	Softening lumps and dissipating nodules
Immature Trifoliate-orange Fruit	95	Regulating *Qi* circulation, expanding chest, eliminating stagnation	Regulating *Qi* circulation
*Paeonia lactiflora* Pall	93	Smoothing liver, relieving pain, nourishing blood, regulating menstruation, antiperspiration	Nourishing blood
Orange seed	91	Regulating *Qi* circulation, dissipating nodules, relieving pain	Regulating *Qi* circulation
Snakegourd Fruit	90	Liberating lung and soothing pharynx, expelling phlegm, discharging pus	Clearing phlegm
Taraxacum	88	Clearing heat, detoxifying, reducing swelling, dissipating nodules, diuresis	Clearing heat and disintoxication
Seaweed	87	Softening lumps, expelling phlegm, dissipating nodules, promoting water metabolism, reducing swelling	Softening lumps and dissipating nodules
Poria cocos	87	Promoting water metabolism, clearing damp, tonifying spleen, tranquilizing mind	Promoting water metabolism
Khumbu	86	Softening lumps, expelling phlegm, dissipating nodules, promoting water metabolism, reducing swelling	Clearing phlegm
Antler	85	Promoting blood circulation, reducing swelling, nourishing kindeys	Nourishing *Yang*-essence
*Glycyrrhiza uralensis* Fisch	85	Nourishing *Qi*, elevating *Yang*-essence, firming surface, stopping sweating, promoting water metabolism, reducing swelling, increasing body fluids, nourishing blood, eliminating stagnation, detoxifying, discharging pus, constricting wound, promoting muscle growth	Nourishing *Qi*
Largehead Atractylodes Rhizome	83	Tonifying spleen, nourishing *Qi,* clearing damp, promoting water metabolism	Nourishing *Qi*
Epimedium	82	Tonifying kidneys, strengthening muscles and bones, expelling wind, clearing damp	Nourishing *Yang*-essence
Borneol	81	Refreshing brain, clearing heat, relieving pain	Refreshing brain
Bupleurum	81	Expelling wind, detoxifying, dissipating nodules, dredging channels and collaterals, relieving pain	Soothing liver
Rhubarb	79	Clearing heat, promoting bowel movement, cooling blood, detoxifying, removing blood stasis	Purgation
Figwort Root	79	Cooling blood, nourishing *Yin*-essence, clearing heat, detoxifying	Nourishing *Yin*-essence
Centipede	78	Expelling wind, detoxifying, dissipating nodules, dredging channels and collaterals, relieving pain	Soothing liver
Honeysuckle	78	Clearing heat, detoxifying, expelling wind	Clearing heat and disintoxication
Angelica Dahurica	77	Relieving external symptoms, expelling wind, relieving pain, refreshing brain, clearing damp, reducing swelling, discharging pus	Relieving external symptoms
Notoginseng	77	Dispelling blood stasis, hemostasis, reducing swelling, relieving pain	Promoting blood circulation and removing blood stasis
*Pinellia ternata*	76	Clearing damp, expelling phlegm	Clearing phlegm
Tangerine leaf	76	Soothing liver, regulating *Qi* circulation, expelling phlegm, detoxifying	Regulating *Qi* circulation
Turtle Shell	75	Nourishing *Yin*-essence, softening lumps, clearing heat, dissipating nodules	Nourishing *Yin*-essence
Forsythia	74	Detoxifying, reducing swelling, dissipating nodules, clearing heat, expelling wind	Clearing heat and disintoxication
Draconis Sanguis	72	Dispelling blood stasis, relieving pain, hemostasis, promoting muscle growth	Promoting blood circulation and removing blood stasis
Herba Hedyotidis	70	Clearing heat, detoxifying, diuresis, reducing swelling, promoting blood circulation, relieving pain	Clearing heat and disintoxication

**Table 3:** Herbal pair and its correlation coefficient.

Herbal pair		Frequency	Correlation coefficient	Herbal pair		Frequency	Correlation coefficient
*Astragalus mongholicus*	*Angelica sinensis*	138	0.3152	Bupleuri Radix	Sedge	80	0.1908
*Angelica sinensis*	Suberect Spatholobus Stem	135	0.3078	Sedge	Seaweed	77	0.1855
Sedge	Aromatic Turmeric Root-tuber	128	0.3028	*Prunella vulgaris* L	Seaweed	77	0.1855
Sedge	*Prunella vulgaris* L	128	0.3028	Bupleuri Radix	Safflower	76	0.1831
*Prunella vulgaris* L	Aromatic Turmeric Root-tuber	121	0.2886	Sedge	*Gleditsia sinensis* Lam	73	0.1756
*Ligusticum wallichii*	*Salvia miltiorrhizae*	118	0.2806	Sedge	Poria cocos	73	0.1756
*Angelica sinensis*	Bupleuri Radix	116	0.2765	Sedge	*Thunberg fritillary* bulb	71	0.1688
Corydalis Rhizoma	Suberect Spatholobus Stem	116	0.2765	Bupleuri Radix	*Prunella vulgaris* L	71	0.1688
*Prunella vulgaris* L	Radix Paeoniae Rubra	109	0.2632	Bupleuri Radix	Unripe orange	71	0.1688
Sedge	Radix Paeoniae Rubra	109	0.2632	*Angelica sinensis*	Sedge	69	0.1624
*Prunella vulgaris* L	Unripe Orange	106	0.2558	*Angelica sinensis*	Peach Pit	69	0.1624
Sedge	Unripe Orange	106	0.2558	Bupleuri Radix	Aromatic Turmeric Root-tuber	67	0.1573
Sedge	Pangolin	99	0.2398	*Prunella vulgaris* L	*Thunberg fritillary* bulb	66	0.1558
Sedge	Vaccariae Semen	99	0.2398	Bupleuri Radix	*Salvia miltiorrhizae*	65	0.1532
*Angelica sinensis*	Peach Pit	98	0.2363	*Angelica sinensis*	Orange seed	65	0.1532
*Prunella vulgaris* L	Pangolin	98	0.2363	*Angelica sinensis*	Aromatic Turmeric Root-tuber	63	0.1488
Sedge	Frankincense	96	0.2321	Bupleuri Radix	*Paeonia lactiflora* Pall	63	0.1488
*Prunella vulgaris* L	Frankincense	96	0.2321	*Angelica sinensis*	*Prunella vulgaris* L	62	0.1462
Prunella vulgaris L	Safflower	95	0.2296	*Angelica sinensis*	*Astragalus mongholicus*	60	0.1426
Radix Achyranthis Bidentatae	Safflower	95	0.2296	*Angelica sinensis*	*Salvia miltiorrhizae*	60	0.1426
Sedge	Suberect Spatholobus Stem	92	0.2223	Bupleuri Radix	Corydalis Rhizoma	58	0.1372
*Prunella vulgaris* L	Suberect Spatholobus Stem	92	0.2223	Curcumae Rhizoma	Seaweed	58	0.1372
Radix Achyranthis Bidentatae	Bupleuri Radix	87	0.2101	Radix paeoniae rubra	*Ligusticum wallichii*	57	0.1346
*Prunella vulgaris* L	*Salvia miltiorrhizae*	87	0.2101	Curcumae Rhizoma	Myrrh	56	0.1312
*Prunella vulgaris* L	Oyster	84	0.2021	Aromatic Turmeric Root-tuber	Corydalis Rhizoma	55	0.1289
Sedge	Oyster	84	0.2021	Aromatic Turmeric Root-tuber	Unripe Orange	55	0.1289
*Prunella vulgaris* L	Taraxacum	83	0.1987	Aromatic Turmeric Root-tuber	Sparganii Rhizoma	53	0.1223
*Prunella vulgaris* L	*Ligusticum wallichii*	81	0.1942	Curcumae Rhizoma	Unripe orange	52	0.1190
*Prunella vulgaris* L	Corydalis Rhizoma	81	0.1942	Unripe orange	Oyster	51	0.1156
Sedge	*Ligusticum wallichii*	81	0.1942	Radix paeoniae rubra	Safflower	50	0.1123
Sedge	Corydalis Rhizoma	80	0.1908	Radix paeoniae rubra	Unripe orange	50	0.1123

**Table 4:** Efficacy combinations of Chinese herbs.

Efficacy combinations	Herbs	Herbs
Herbal pairs for promoting blood circulation	*Salvia miltiorrhizae*	Curcumae Rhizoma, Radix paeoniae rubra, Vaccariae Semen, frankincense, Safflower, Sparganii Rhizoma, *Ligusticum wallichii*, Corydalis Rhizoma, Peach Pit, Motherwort
	Curcumae Rhizoma	Radix paeoniae rubra, Vaccariae Semen, Frankincense, Safflower, Sparganii Rhizoma, Myrrh, *Ligusticum wallichii*, Corydalis Rhizoma, Peach Pit, Tree Peony Bark, Motherwort, Rhubarb, Notoginseng, Suberect Spatholobus Stem
	Radix paeoniae rubra	Vaccariae Semen, Frankincense, Safflower, Sparganii Rhizoma, Myrrh, *Ligusticum wallichii*, Corydalis Rhizoma, Peach Pit, Tree Peony Bark
	Vaccariae Semen	Frankincense, Safflower, Sparganii Rhizoma, Myrrh, *Ligusticum wallichii*, Corydalis Rhizoma, Peach Pit, Tree Peony Bark, Motherwort, Suberect Spatholobus Stem, Leech, Rhubarb, notoginseng, Draconis Sanguis
	Safflower	Sparganii Rhizoma, Myrrh, *Ligusticum wallichii*, Corydalis Rhizoma, Peach Pit, Tree Peony Bark, Motherwort, Notoginseng, Suberect Spatholobus Stem
	Sparganii Rhizoma	Myrrh, *Ligusticum wallichii*, Corydalis Rhizoma, Peach Pit, Tree Peony Bark, Bupleurum, Bupleuri Radix, Motherwort, Rhubarb, Notoginseng, Draconis Sanguis, Suberect Spatholobus Stem
	*Ligusticum wallichii*	Corydalis Rhizoma, Peach Pit, Tree Peony Bark, Motherwort, Rhubarb, Notoginseng, Suberect Spatholobus Stem
Herbal pairs for promoting *Qi* circulation	Unripe orange	Orange seed, Orange peel, Aucklandiae Radix, Immature Trifoliate-orange Fruit, Litchi Semen
Promoting *Qi* circulation + promoting blood circulation	Sedge	*Salvia miltiorrhizae*, Radix paeoniae rubra, Curcumae Rhizoma, Vaccariae Semen, Frankincense, Safflower, Sparganii Rhizoma, Myrrh, *Ligusticum wallichii*, Corydalis Rhizoma, Peach Pit, Tree Peony Bark
	Unripe orange	*Salvia miltiorrhizae*, Curcumae Rhizoma, Radix paeoniae rubra, Vaccariae Semen, Frankincense, Safflower, Sparganii Rhizoma, Myrrh, *Ligusticum wallichii*, Corydalis Rhizoma, Peach Pit, Motherwort, Rhubarb, Suberect Spatholobus Stem
	Orange peel	Radix paeoniae rubra, Curcumae Rhizoma, Vaccariae Semen, Safflower, *Ligusticum wallichii*
	Aucklandiae Radix	Radix paeoniae rubra, Curcumae Rhizoma, Vaccariae Semen, Safflower, Motherwort, *Ligusticum wallichii*, *Salvia miltiorrhizae*
	Chinaberry fruit	Radix paeoniae rubra, Vaccariae Semen
	Orange seed	Vaccariae Semen, *Salvia miltiorrhizae*, Curcumae Rhizoma, Radix paeoniae rubra, Safflower, *Ligusticum wallichii*
	Aromatic Turmeric Root-tuber	*Salvia miltiorrhizae*, Curcumae Rhizoma, Radix paeoniae rubra, Vaccariae Semen, Frankincense, Safflower, Sparganii Rhizoma, Myrrh, *Ligusticum wallichii*, Corydalis Rhizoma
	Immature Trifoliate-orange Fruit	Vaccariae Semen, Safflower
	Litchi Semen	Vaccariae Semen, Safflower, *Ligusticum wallichii*
Nourishing blood + promoting blood circulation	*Angelica sinensis*	Sparganii Rhizoma, Myrrh, *Ligusticum wallichii*, *Salvia miltiorrhizae*, Corydalis Rhizoma, Curcumae Rhizoma, Radix paeoniae rubra, Vaccariae Semen, Peach Pit, Frankincense, Safflower, Suberect Spatholobus Stem, Notoginseng, Tree Peony Bark, Motherwort, Radix Achyranthis Bidentatae
	*Paeonia lactiflora* Pall	*Salvia miltiorrhizae*, Curcumae Rhizoma, Radix paeoniae rubra, Vaccariae Semen, Safflower, Sparganii Rhizoma, Myrrh, *Ligusticum wallichii*, Corydalis Rhizoma, Peach Pit, Tree Peony Bark, Motherwort, Rhubarb, Notoginseng, Draconis Sanguis, Suberect Spatholobus Stem
Nourishing blood + promoting *Qi* circulation	*Angelica sinensis*	Sedge, Aromatic Turmeric Root-tuber, Unripe orange, Orange seed, Orange peel, Aucklandiae Radix, Chinaberry Fruit
	*Paeonia lactiflora* Pall	Unripe orange, Orange seed, Orange peel, Aucklandiae Radix, Chinaberry Fruit, Immature Trifoliate-Orange Fruit, Litchi Semen
Reducing swelling/dissipating nodules/discharging pus + promoting blood circulation	*Prunella vulgaris* L	*Salvia miltiorrhizae*, Curcumae Rhizoma, Radix paeoniae rubra, Vaccariae Semen, Frankincense, Safflower, Sparganii Rhizoma, Myrrh, *Ligusticum wallichii*, Corydalis Rhizoma, Peach Pit, Tree Peony Bark, Motherwort
	Pangolin/*Gleditsia sinensis* Lam	Curcumae Rhizoma, Radix Paeoniae Rubra, Vaccariae Semen, *Salvia miltiorrhizae*
	*Angelica dahurica*	Safflower
	*Gleditsia sinensis* Lam	*Ligusticum wallichii*
Soothing liver + promoting blood circulation	Bupleuri Radix	Sparganii Rhizoma, Myrrh, *Salvia miltiorrhizae*, *Ligusticum wallichii*, Curcumae Rhizoma, Corydalis Rhizoma, Radix paeoniae rubra, Vaccariae Semen, Safflower, Peach Pit,
Soothing liver + promoting *Qi* circulation	Bupleuri Radix	Sedge, Unripe orange, Orange seed
Soothing liver + softening lumps and dissipating nodules	Bupleuri Radix	Snakegourd Fruit, *Prunella vulgaris* L, Oyster, Seaweed, *Thunberg fritillary* bulb, Khumbu
Promoting *Qi* circulation + softening lumps and dissipating nodules	Sedge	*Prunella vulgaris* L, Oyster, Seaweed, *Thunberg fritillary* bulb, Khumbu
	Unripe orange	Oyster, *Thunberg fritillary* bulb
	Aromatic Turmeric Root-tuber	Oyster, Seaweed, *Thunberg fritillary* bulb
Reducing swelling/dissipating nodules + promoting *Qi* circulation	*Prunella vulgaris* L	Aromatic Turmeric Root-tuber, Unripe orange, Orange seed, Orange peel, Aucklandiae Radix, Chinaberry Fruit
Promoting *Qi* circulation + reducing swelling/discharging pus	Sedge	Pangolin, *Gleditsia sinensis* Lam, *Astragalus mongholicus*
	Unripe Orange, Aromatic Turmeric Root-tuber	Pangolin, *Gleditsia sinensis* Lam
Promoting *Qi* circulation + tonifying spleen/promoting water metabolism/clearing damp	Sedge, Unripe orange	Poria cocos, Largehead Atractylodes Rhizome

**Table 5:** Herbal core combinations.

Bupleuri Radix	Orange Peel	Sedge	*Ligusticum wallichii*	Radix Paeoniae Rubra
Peach Pit	Safflower	*Angelica sinensis*	*Ligusticum wallichii*	Radix Paeoniae Rubra
Peach Pit	Safflower	Radix Achyranthis Bidentatae	*Angelica Sinensis*	Bupleuri Radix
Unripe orange	Salviae	*Ligusticum wallichii*	Motherwort	Aucklandiae Radix
*Salvia miltiorrhizae*	*Prunella vulgaris* L	Unripe orange	*Ligusticum wallichii*	Radix Paeoniae Rubra
*Prunella vulgaris* L	Sedge	Aromatic Turmeric Root-tuber	Corydalis Rhizoma	Suberect Spatholobus Stem
*Angelica sinensis*	Largehead Atractylodes Rhizome	Radix Paeoniae Rubra	Poria cocos	
*Angelica sinensis*	Raw Rehmannia root	Ligusticum wallichii	Radix Paeoniae Rubra	
Peach Pit	Tree Peony Bark	Frankincense	Myrrh	
*Ligusticum wallichii*	Bupleuri Radix	Unripe Orange	Sedge	
Peach Pit	Safflower	Platycodon Grandiflorum	Radix Paeoniae Rubra	
Orange Peel	*Ligusticum wallichii*	*Paeonia lactiflora* Pall	Radix Paeoniae Rubra	
Angelica Sinensis	Raw Rehmannia Root	*Ligusticum wallichii*	Radix Paeoniae Rubra	
Bupleuri Radix	*Paeonia lactiflora* Pall	*Angelica sinensis*	*Glycyrrhiza uralensis* Fisch	
Bupleuri Radix	*Angelica sinensis*	Largehead Atractylodes Rhizome	*Paeonia lactiflora* Pall	
Largehead Atractylodes Rhizome	Poria Cocos	Paeonia Lactiflora Pall	*Glycyrrhiza uralensis* Fisch	
*Salvia miltiorrhizae*	*Gleditsia sinensis* Lam	Vaccariae Semen		
Sedge	Unripe Orange	*Salvia miltiorrhizae*		
Immature Trifoliate-orange Fruit	Radix Paeoniae Rubra	Sedge		
Bupleuri Radix	Immature Trifoliate-Orange Fruit	*Ligusticum wallichii* Grandiflorum		
*Ligusticum wallichii*	Radix Achyranthis Bidentatae	Radix Paeoniae Rubra		
Bupleuri Radix	Immature Trifoliate-Orange Fruit	*Glycyrrhiza uralensis* Fisch		
Bupleuri Radix	*Angelica sinensis*	*Paeonia lactiflora* Pall		
Peach Pit	Suberect Spatholobus Stem	*Salvia miltiorrhizae*		
Bupleuri Radix	Poria cocos	Largehead Atractylodes Rhizome		
*Angelica sinensis*	*Astragalus mongholicus*	*Ligusticum wallichii*		
Sparganii Rhizoma	Safflower	Radix Paeoniae Rubra		
*Prunella vulgaris* L	*Thunberg fritillary* bulb	Unripe Orange		
Pangolin	*Gleditsia sinensis* Lam	*Thunberg fritillary* bulb		
Orange Peel	Poria Cocos	Largehead Atractylodes Rhizome		
Pangolin	Vaccariae Semen	*Gleditsia sinensis* Lam		

Verification of the statistical significance of the results:

1. Significance testing of occurrence of high frequency herbs: The chi-square test was used to verify the frequency of occurrence of high frequency herbs. The reliability of frequency estimation was analyzed by confidence intervals to avoid small-sample errors.
2. Robustness of cluster analysis: The sensitivity of parameters in the entropy clustering (such as the selection of threshold δ) was evaluated. The stability of core combinations was observed by adjusting the parameters multiple times and repeating the clustering process. The silhouette coefficient and cluster stability index were used to evaluate the clustering quality by measuring the average intra-cluster distance and the inter-cluster distance.
3. Quantitative evaluation of association rules: The support and confidence of herbal pairs and core combinations were calculated, and the strength of the association was determined using the lift metric. The a priori algorithm and Frequent-Pattern-Growth (FP-Growth) algorithm were used for 2-way verification of the statistical significance for high frequency combinations.
4. Time-trend analysis: The data of the patents from different time periods (such as every 10 years) was analyzed to determine the stability of high frequency herbal combinations and exclude historical biases.

## 4. Discussion

### 4.1. Promoting blood circulation and removing stasis are basic mechanisms of TCM treatments for breast hyperplasia

The results of research studies have shown the importance of promoting blood circulation and removing stasis in the treatment of breast hyperplasia: Efficacy frequency (Table 1, promoting blood circulation 1116, removing blood stasis 507), Chinese herb types (Table 2, Aromatic Turmeric Root-tuber 99, *Salvia miltiorrhizae* 97, Curcumae Rhizoma 96, Vaccariae Semen 83, pangolin 82, frankincense 78, Sparganii Rhizoma 77, myrrh 77, *Ligusticum wallichii* 71, Corydalis Rhizoma 71, Peach Pit 48, Tree Peony Bark 38, Motherwort 33, Notoginseng 27, Draconis Sanguis 24, Suberect Spatholobus Stem 23). Herbal pairs are the combination of 2 kinds of herbs with the effect of promoting blood circulation and removing blood stasis (Table 4, 73). The combination of several herbs to promote blood circulation or remove blood stasis was reflected in the core combination (Table 5, Peach Pit + Tree Peony Bark + frankincense + myrrh, Sparganii Rhizoma + Radix paeoniae rubra + Safflower).

Herbs with promoting blood circulation and removing blood stasis effects are often used to treat phlegm and fire stasis, accumulation of lumps caused by *Qi* stagnation or blood stasis (the main pathological manifestations of MGH), which mainly occur in liver, gallbladder or kidney channels. These herbs play a role in treating MGH by promoting blood circulation, removing blood stasis, promoting *Qi* movement, and relieving pain. Their main pharmacological effects are as follows.

#### 4.1.1. Homeostasis of the endocrine system

MGH is an endocrine system disorder mainly caused by abnormal hormone secretion leading to a hormonal imbalance. MGH patients have significantly increased estradiol (E_2_) levels and significantly decreased P levels, which results in abnormal proliferation of mammary epithelial cells and fibrous tissues. Excessive prolactin (PRL) can increase E_2_, and gonadotropin-releasing hormone can promote pituitary secretion of luteinizing hormone (LH) and follicular stimulating hormone (FSH), that in turn promote ovarian secretion of E_2_ and P, which negatively regulate the hypothalamus and pituitary gland, thus maintaining the endogenous balance and normal physiological function of the female endocrine system.^[3,4]^

The herbs that promote blood circulation and remove blood stasis exert their breast protective effects by regulating the levels of various hormones, including E_2_, P, PRL, gonadotropin-releasing hormone, LH, FSH, etc.

#### 4.1.2. Restoring the normal expression of the hormone receptor

E_2_ and P play an important biological role in the balance between their estrogen receptors (ERs), namely estrogen receptor 1 (ESR1) and estrogen receptor 2 (ESR2) and progesterone receptor (PGR/PR). Changes in the expression of E_2_ or P stimulate the overexpression of ESR1 and PR in breast tissue, which eventually lead to the loss of the breast tissue protective function.^[5]^ PRL binds to prolactin receptors (PRLRs) of breast epithelial cells, which leads to the activation of intracellular signaling pathways, such as Janus kinase 2/signal transducers and transcriptional activators 5, and PI3K/Akt (phosphatidyl inositol 3 kinase/AKT serine/threonine kinase 1), that regulate the proliferation and differentiation of breast epithelial cells, maintain the normal function of breast glands. However, overactivation of related pathways by PRLRs can lead to an imbalance between proliferation and apoptosis. Herbs for promoting blood circulation and removing blood stasis can prevent breast hyperplasia by regulating the expression of endocrine hormones and their receptors, such as ESR1, PGR, PRLRs.

#### 4.1.3. Inhibiting the proliferation of mammary epithelial cells

Herbs with promoting blood circulation and removing blood stasis effects improve MGH by increasing the expression of proapoptotic proteins or decreasing the expression of antiapoptotic proteins. The B-cell lymphoma 2 (BCL2) apoptosis regulator family of proteins and cysteine aspartic protease (caspase) family of proteins play an important regulatory role in the process of apoptosis.^[6]^ The antiapoptotic proteins of the BCL2 protein family include BCL2 and BCL2 like 1 (BCL2L1), and the main proapoptotic protein of the BCL2 family is BCL2 associated X, apoptosis regulator (BAX). The ratio of antiapoptotic proteins to proapoptotic proteins can be changed through treatment with herbs by activating blood circulation and removing blood stasis, which can ultimately regulate cell proliferation or apoptosis. The herbs play the role in regulating cell apoptosis by adjusting BCL2/BAX, thereby activating the caspase cascade and the expression of caspase 3 (CASP3).^[7]^ The protective mechanism against breast hyperplasia involves releasing cytochrome *c*, downregulating antiapoptotic proteins αB-lens protein and BCL2, and promoting the expression of apoptotic protein, such as caspases 3, caspases 8, caspases 9, BAX, and tumor protein 53.^[8]^

#### 4.1.4. Inhibiting the formation of new blood vessels in the lesion

Vascular endothelial growth factor-A (VEGFA) can promote the growth and proliferation of endothelial cells and increase the permeability of blood vessels.

VEGFA and fibroblast growth factor 1 are highly expressed in patients with breast hyperplasia, and can stimulate angiogenesis through common mechanisms in vivo and in vitro, and are important indicators of angiogenesis.^[9]^ Reducing the expression of VEGFA and fibroblast growth factor 2 (FGF2) in serum and proliferative tissues is an effective way to treat MGH.^[10]^ Herbs with promoting blood circulation and removing blood stasis effects can inhibit the transfer of vascular endothelial growth factors mentioned above and reduce vascular permeability, thus inhibiting the formation of new blood vessels, indirectly inhibiting the enlargement of lesion sites, and improving microvascular density, thereby alleviating MGH related symptoms.

#### 4.1.5. Improving hemorheology and hemodynamics

TCM assumes that the main cause of MGH lies in *Qi* stagnation, sputum coagulation and blood stasis, which accumulate in breasts for a long time. The initial cause is the imbalance of blood “perfusion” and “flow” in the mammary tissue, which leads to blood stasis and causes the blood to be in a state of high viscosity or coagulated. Since herbs with promoting blood circulation and removing blood stasis effects can promote blood circulation, remove blood stasis, clear phlegm, eliminate edema, and promote *Qi* circulation, they can improve the hemorheological state and restore blood circulation by adjusting the balance of “perfusion” and “flow,” and play a protective role against MGH.^[11]^

### 4.2. Soothing the liver and regulating Qi play an important role in the treatment of MGH

The *Foot Jue-yin* liver meridians run through the breast area. The liver, as a resolute organ, prefers to be relaxed state and dislikes restrain. It is believed in TCM that MGH has a close relationship with emotional depression. Emotional depression and anger may lead to liver *Qi* stagnation, which blocks blood circulation in the breasts and related meridians, causing swelling and pain. Therefore, soothing the liver, relieving depression, promoting the circulation of *Qi*, and alleviating pain are important ways of treating breast hyperplasia by TCM.

It is believed in TCM that *Qi* is the driving force of human life activities. If the function of *Qi* is normal, the blood runs normally, and the diet metabolism is normal. Emotional abnormalities can very easily induce *Qi* stagnation, resulting in spittoon coagulation, blood stasis, and the formation of lumps, which manifests as a knot in the chest and leads to MGH. According to TCM theory, “lack of circulation of *Qi* and blood causes pain,” pain and lumps are the main clinical manifestations of breast hyperplasia. Therefore, a basic treatment for breast hyperplasia is to eliminate lumps by activating *Qi* circulation.

The frequency of herbs with liver soothing effect was 526 (Table 1). Among the high frequency herbs, there are 3 herbs with liver soothing effect (Table 2; Bupleuri Radix 172, Scorpion 34, Centipede 29). The frequency of herbs with activating *Qi* circulation effect was 854. Among the high frequency herbs, there are 8 herbs with activating *Qi* circulation effect (Table 2; Sedge 140, Unripe orange 86, Orange seed 48, Orange peel 40, Aucklandiae Radix 39, Chinaberry Fruit 37, Tangerine leaf 26, Immature Trifoliate orange Fruit 24). The combined use of herbs with activating *Qi* circulation is reflected in the drug pairs (Table 4, Unripe orange + Orange seed/Orange peel/Aucklandiae Radix/Immature Trifoliate orange Fruit/Litchi Semen). The combination of herbs with liver soothing effect and herbs with activating *Qi* circulation effect is reflected in herbal pairs and herbal core combination (Tables 3–5; Bupleuri Radix + Sedge, Bupleuri Radix + Unripe orange, Bupleuri Radix + Orange seed, Bupleuri Radix + Unripe orange + Sedge).

#### 4.2.1. Pharmacological effects of herbs with liver soothing properties in the treatment of breast hyperplasia

The expression of angiogenic factors (e.g., VEGFA, FGF2) is closely related to the occurrence and development of breast hyperplasia. VEGFA can not only increase the permeability of vascular endothelial cells, but also induce the development of new mechanisms and new blood vessels by increasing vascular permeability. FGF2 is a direct inducer of angiogenesis, which can promote the regeneration of epidermal cells and endothelial cells, induce the proliferation of vascular endothelial cells and detach them from the basement membrane, which in turn induce chemotaxis of endothelial cells towards breast variant cells.

Herbs with liver soothing effect inhibit the proliferation of breast cells by downregulating the expression of angiogenic factors (VEGFA and FGF2), thereby improving pituitary and ovarian function, regulating endocrine function, and improving microcirculation and local blood circulation.^[12]^

Herbs with liver soothing effect can not only promote the metabolism of E_2_ in the liver and reduce its promotion of the expression of ER messenger ribonucleic acid (mRNA), but also reduce the sensitivity of mammary tissue to E_2_, enhance P secretion, reduce blood viscosity, improve blood circulation, and promote swelling and fiber absorption.

Soothing the liver can adjust the hypothalamic–pituitary–adrenal axis, adjust the cortisol level, and thus alleviate the chronic stress state, thereby improving the abnormal mood of MGH patients, so as to achieve the purpose of healing.^[13–15]^

#### 4.2.2. Pharmacological effects of herbs with Qi-regulating properties on MGH

Herbs with *Qi*-regulating properties have obvious therapeutic effects on MGH, which are manifested in removing proliferative tissue, reducing the number of mammary gland lobules and acini, reducing vacuolar degeneration, hyperplasia and shedding of glandular epithelial cells.

When the E_2_ level increases and P level decreases, the breast tissue is stimulated by E_2_, breast ducts and lobules proliferate, tissue water and sodium are retained, causing lobular interstitial edema.^[16]^

The lack of P regulation and protection leads to MGH or incomplete recovery, which result in varying degrees of hyperplasia of the breast ductal epithelium and fibrous tissue, as well as cyst formation in distal glandular ducts or acini.

Herbs with *Qi*-regulating effects can decrease serum E_2_ level and increase P content, and the efficacy is positively correlated with the dose.^[17]^

The overexpression of ESR1, ESR2 and PGR can aggravate the occurrence and development of MGH, and ESR1/2 signaling can regulate the growth, development and apoptosis of mammary gland cells.^[18,19]^

The mammary gland is a target organ of E_2_ and P, and the proliferation of the mammary gland is closely related to the disorder of E_2_ and P production, as well as their receptors.^[20]^

*Qi*-regulating herbs can significantly reduce the protein expression levels of ESR1, ESR2 and PGR in rat mammary tissue. The thymus and spleen are important endocrine organs. E_2_ can directly act on the ESR1 and ESR2 of thymocytes, induce programmed cell death in thymocytes, which leads to the reduction of the proportion of lymphocytes in the thymic cortex and medulla, the degeneration of the thymus, and a decrease in the organ coefficient.^[21]^

The spleen is the organ where lymphocytes reside and proliferate, as well as become activated to perform immune functions and generate specific immune responses. E_2_ can diminish splenic medullary hematopoiesis, and decreased the number of lymphocytes and macrophages.^[22]^

MGH patients have immunosuppression, immunomodulatory dysfunction, and immune response disorders, so that the immune system cannot effectively identify, suppress and remove the diseased cells.

Herbs with *Qi*-regulating properties can significantly increase the weight index of the thymus and spleen in MGH rats, protect against the damage of immune organs caused by an abnormal increase in E_2_ levels in a pathological state.^[23]^

Herbs with *Qi*-regulating properties can reduce the number of acini in MGH tissue, leading to acinar atrophy and duct lumen minimization, which effectively limit hyperplasia. Herbs with *Qi*-regulating properties can reduce the E_2_ level, increase the P level, reduce the expression of ESR1 and ESR2 at the molecular level, which are used to treat MGH by regulating the endocrine system and reducing the expression levels hormone receptors.

### 4.3. Herbs with heat-clearing and detoxification properties can effectively reduce swelling and pain MGH patients

The results revealed that the herbs with heat-clearing and detoxification properties have a large number in MGH effective patents: herbs with heat-clearing properties (985, Table 1), herbs with detoxification properties (546, Table 1), among the high frequency herbs, there are 4 herbs with heat-clearing and detoxification properties (taraxacum 73, honeysuckle 29, forsythia 24, Herba Hedyotidis 21).

According to the medical theory of the Zhuang ethnic minority in China, there are 3 important avenues (heaven, earth, and human) and 2 pathways (blood transportation, and information sensing) in the human body. An unobstructed state is important for the 3 important avenues and 2 pathways, which results in the harmony of *Qi* and blood, equilibrium of *Yin* and *Yang*, and normal function of organs.^[24]^

“Poison” is a general term in the medical theory of the Zhuang ethnic minority in China. Whether the virus causes a disease after invading human body depends on the virulence strength and the human health status. If the virulence is too strong or the physical condition of the host is poor, the body energy and evil poison compete with each other, but the body energy is powerless against the evil poison, thus the 3 important avenues (heaven, earth, human) and 2 pathways (blood transportation, information sensing) are blocked by the evil poison, which results in diseases. MGH is mainly caused by the invasion of wind poison, cold poison, wet poison, heat poison and other toxic pathogens, which stagnate in the viscera and bone marrow, and obstruct the 3 important avenues and 2 pathways. The accumulation of evil poison leads to heat and swelling pain; thus the ways of clearing heat and detoxification are common treatments for MGH.

Proliferating cell nuclear antigen (PCNA) is a processivity factor for the deoxyribonucleic acid (DNA) polymerase responsible for the synthesis of the DNA leading strand in DNA replication, which is essential for timely and faithful replication of DNA genome, The expression of PCNA is related to cell cycle, and is the weakest in the static stage, but active in the proliferating and transmutation stages. The PCNA index is one of the important indexes to evaluate the proliferative state of the cell.^[25]^

One of the pathological manifestations of MGH is the hyperplasia of catheter epithelial cells and interstitial fibrous tissue. The hyperplastic breast tissue is in a highly active proliferation state, and the expression of PCNA is significantly increased. Herbs with heat-clearing and detoxification properties can inhibit the PCNA protein synthesis and gene expression by preventing cell entry into the S-phase and inducing the DNA synthesis, which reduce the E_2_ content, inhibit the abnormal stimulation of breast tissue, and prevent and treat MGH.^[26]^ BCL2 acts as a cell survival protein and latent suppressor to inhibit apoptosis.^[27]^ During the occurrence and development of MGH, BCL2 is the important control point that affects the epithelial cells of milk gland ducts.^[28,29]^ Herbs with detoxification properties induce apoptosis by suppressing BCL2 expression, and reducing the proliferation of breast tissue cells. Detoxifying herbs can activate and enhance the function of T lymphocytes, attach to the milk gland tissue by lymphocyte networks and capillary vascular networks, kill hyperplastic breast cells through the FAS ligand (FASL)/FAS death pathway. In the course of treating MGH, herbs with detoxification properties can regulate the apoptotic signaling pathway of FASL- and FAS-positive cells between immune cells and breast organization, increase the level of fatty acid synthase (FAS) protein in breast tissue, reduce the degree of hyperplasia degree of breast tissue of MGH rats.^[30]^

### 4.4. Adjuvant treatment of MGH with other TCM herbs

#### 4.4.1. Reducing lumps, dispersing knots, softening hard masses

The results of research studies have shown that the herbs’ frequency for reducing lumps and dispersing knots was 938 (Table 1), and the herbs’ frequency for softening hard masses was 620 (Table 1). Among the high frequency herbs, there were 2 herbs for reducing lumps, softening hard mass and dissolving knots (Table 2, *Prunella vulgaris* L 126, and Oyster 74). These herbs are used to aid major herbs in reducing lumps and pain, and relieving symptoms.

#### 4.4.2. Eliminating phlegm, resolving stagnation and removing pus

The results of research studies have shown that the herbs’ frequency of eliminating phlegm was 440 (Table 1), that of resolving stagnation was 415 (Table 1), and that of removing pus was 353 (Table 1). Among the high frequency herbs, there were 4 expectorant herbs in the forefront (Table 2, *Snakeg ourd Fruit* 75, *Gleditsia sinensis* Lam 61, *Thunberg fritillary* bulb 61, and Khumbu 56).

Sputum stagnation is an important etiology and pathogenesis of MGH. Exogenous pathogenic factors, emotional injury and diet fatigue, result in the 5 viscera system (mainly lung, spleen, and kidney) and *Sanjiao-system* gasification dysfunction, which cause phlegm to accumulate in the chest and cause breast hyperplasia. Or due to liver and kidney insufficiency, disorder of *Chong* and *Ren* meridians, deficiency of *Yang*, phlegm and dampness knot accumulate in the breast over time to form a lump. Phlegm turbidity as visible *Yin* evil, adsorptive and sticky can affect the movement of *Qi* and blood, lead to a lingering disease being transformed into heat evil, which usually erodes the normal breast tissue and forms an abscess. Therefore, resolving phlegm and eliminating its accumulation, reducing swelling and draining pus are important measures for preventing and treating breast hyperplasia.

#### 4.4.3. Tonifying Qi and blood, nourishing Yin, generating muscle

Among the high frequency herbs, *Angelica sinensis* (150), and *Paeonia lactiflora* Pall (79) are herbs for enriching blood, while *Astragalus mongholicus* (86), *Glycyrrhiza uralensis* Fisch (46), and *Largehead Atractylodes Rhizome* (45) are herbs for invigorating *Qi,* and *Figwort Root* (29), and *Turtle Shell* (25) are herbs for nourishing *Yin.*

MGH often manifests as the loss of positive energy and accumulation of pathological products. *Qi* stagnation, phlegm turbidness, and blood stasis accumulate in the breast tissue. Also, on the 1 hand, MGH patients show symptoms of breast swelling and pain. On the other hand, the above pathological products will further block the normal production and metabolism of *Qi* and blood, which results in *Qi* and blood deficiency. Without the nourishment of fine nutrients, such as *Qi* and blood, it is difficult to heal the pathological tissue. Therefore, the combination of tonifying deficiency and expelling pathogenic factors is a commonly used approach for treating MGH, while nourishing *Yin*, generating muscle, tonifying *Qi* and blood, are commonly used approaches for treating MGH.

### 4.5. Medication rules in herbal pairs and core combinations

Medical pair and core combination of herbs are commonly used and relatively fixed forms of herbal combination in clinical practice, and are the smallest prescription unit, which have the basic function of prescriptions and show the overall efficacy of prescriptions. The study on its compatibility combinations is of great value to reveal the compatibility laws and scientific connotations of prescriptions.

#### 4.5.1. *Activating blood circulation + activating Qi circulation*

Among the effective patents of herbal prescriptions, the combinations of promoting *Qi* circulation and promoting blood circulation occur more frequently (Table 3; 58 pairs in total). The herbal pairs with higher frequency and correlation coefficient are shown in Table 4 (Sedge + *Salvia miltiorrhizae*, Sedge + Curcumae Rhizoma, Sedge + *Vaccariae Semen*, Radix paeoniae rubra + *Ligusticum wallichii*, Radix paeoniae rubra + Unripe orange …). The core combinations for promoting blood circulation and promoting *Qi* circulation are shown in Table 5 (*Salvia miltiorrhizae* + *Gleditsia sinensis* Lam + Vaccariae Semen, Sedge + Unripe orange + *Salvia miltiorrhizae*).

*Qi* and blood are the basic nutrients of the human body, and have the relationship of interdependence, mutual breeding and utilization. *Qi* can produce blood, control and promote blood circulation in the meridians, while blood can generate *Qi* and have the movement of *Qi.* The close physiological relationship between the 2 results in the pathological occurrence of a lesion on 1 side quickly affecting the other, poor circulation of *Qi* will soon produce blood stasis, while blood stasis further blocks the movement of *Qi,* which causes lump stagnates in the chest to produce MGH. Accordingly, promoting blood circulation in combination with promoting *Qi* circulation is effective and rapid in treating MGH, and the pharmacological mechanism is as follows.

The herbal combination of promoting blood circulation and promoting *Qi* circulation can improve blood rheology and local microcirculation.^[31]^ Patients with *Qi* stagnation and blood stasis often exhibit local microcirculation disorders, which is the “*pain caused by obstruction*” in TCM theory. The herbal combination of promoting blood circulation and promoting *Qi* circulation improves microcirculation by expanding blood vessels, providing blood and oxygen to local rigid tissues, reducing hematocrit and whole blood viscosity (WBV), improving the state of thick, sticky and condensed blood, thereby improving hemorheology.^[32]^

Activating blood circulation in combination with activating *Qi* circulation can significantly increase the mesenteric microvascular caliber, reduce the number of capillary network intersection points, and decrease blood flow velocity. By reducing the degree of red blood cell aggregation, blood rheology is improved, blood viscosity and whole blood low shear viscosity are reduced, and blood viscosity under the influence of excluding hematocrit is decreased. By increasing the number of platelets and antiplatelet aggregation, blood viscosity is reduced.

The activity of super oxide dismutase (SOD) in MGH tissue clearly decreases, while the content of the lipid peroxidation metabolite malondialdehyde (MDA) increases. The combination of promoting blood circulation and promoting *Qi* circulation can increase SOD activity, reduce MDA content, which have antioxidant effects and reduce the damage caused by free radicals.

#### 4.5.2. *Supplementing blood + promoting blood circulation*

Among the effective patents of herbal prescriptions for preventing and treating breast hyperplasia, the combination of nourishing blood and promoting blood circulation is widely used (Table 3; 31 pairs in total). The herbal pairs with higher frequency and correlation coefficient are shown in Table 4 (*Angelica sinensis* + *Curcumae Rhizoma* (06, 0.2953), *Paeonia lactiflora Pall* + *Safflower* (78, 0.2635), *Angelica sinensis* + *Vaccariae Semen* (83,0.2787), *Angelica sinensis* + *Peach Pit* (50, 0.2189), *Angelica sinensis* + *Salvia miltiorrhizae* (45, 0.2109)…).

Supplementing blood and promoting blood circulation are mainly aimed at the type of nutrient deficiency and excess of pathogenic factors in MGH, namely the type of blood deficiency and stasis. Blood deficiency refers to insufficient blood storage, and results in a decrease in the nutritional and nourishing function of blood, which lead to dysfunction of organs and tissues due to loss of nourishment. The dysfunction of the liver in storing blood and regulating blood volume leads to abnormal blood flow, blood leaves the meridians and remains outside the veins, which causes blood stasis. Blood stasis does not go, new blood is not produced, so the cycle is repeated, which is the type of blood deficiency and blood stasis obstruction in breast hyperplasia. Patients usually have breast lumps, pain like acupuncture, pale face, dry and rough skin, withered hair, brittle claws dim vision, numbness of hands and feet, poor flexion and extension of limbs, palpitations, insomnia, dreaminess, forgetfulness.

Herbs that can nourish blood and are used to treat blood deficiency are called blood tonics. It is mild in nature, sweet in taste, and belongs to the liver, spleen, and kidney meridians. The pharmacological effects of preventing and treating MGH are as follows: improve bone marrow hematopoietic function; resistance to hypoxia; dilating coronary artery, reducing myocardial oxygen consumption, increasing myocardial blood flow; nourishing blood and liver, reducing serum transaminases, increasing liver glycogen, and improving liver microcirculation.

Blood stasis is related to various pathological factors, including blood viscosity, disorders of microcirculation and blood circulation, platelet activation and adhesion aggregation, abnormal tissue cell metabolism and immune function metabolism, etc. Herbs that have the effects of unblocking blood vessels, dispelling blood stasis, and treating blood stasis blockages are called blood activating and stasis resolving herbs. These herbs have a relatively mild nature (soft nature, slightly cold, or slightly warm), enter 3 meridians (heart, liver, and spleen). They have the functions of promoting blood circulation, relieving pain, regulating menstruation, removing blood stasis and removing ecchymosis spots. The pharmacological action of herbs for promoting blood circulation and resolving blood stasis in the treatment of MGH are as follows: improving blood rheology and preventing thrombosis; improving microvascular status and blood flow; reducing the permeability of capillaries, reducing or clearing blood leakage around microvessels; reducing serum total cholesterol, neutral fat, and β-lipoprotein; and improving hemodynamics, dilating peripheral blood vessels, and increasing organ blood flow.

#### 4.5.3. *Reducing swelling/dissipating nodules/discharging pus + promoting blood circulation*

Among the effective patents’ herbal pairs, there were many herbs for reducing swelling, dispersing nodules, and purulent drainage combined with herbs for promoting blood circulation (Table 3; 19 pairs in total). The herbal pairs with higher frequency and correlation coefficient are shown in Table 4 (*Prunella vulgaris* L + Radix paeoniae rubra (89, 0.2876), *Prunella vulgaris* L + frankincense (82, 0.2762), *Prunella vulgaris* L + Safflower (78,0.2653), *Prunella vulgaris* L + Sparganii Rhizoma (77, 0.2621), *Prunella vulgaris* L + myrrh (77, 0.262), *Prunella vulgaris* L + *Ligusticum wallichii* (71, 0.2512)…).

In TCM theory it is believed that blood is the carrier of *Qi*, and stagnant blood caused by poor blood circulation can result in *Qi* stasis, prolonged *Qi* stagnation and blood stasis affect the normal metabolism of water, which creates a state of *Qi* stagnation, blood stasis, phlegm turbidity and water dampness condensation. Affected by this state for a long time, it produces heat toxicity and lumps. When they accumulate in the chest, it is MGH, which burns the muscles and meridians and transform into pus. Therefore, the combination of promoting blood circulation and reducing swelling, dispersing nodules, expelling pus is a commonly used method in TCM for treating breast hyperplasia.

Chinese herbs for promoting blood circulation and dispersing nodules can effectively improve breast blood circulation, reduce blood viscosity, alleviate pain, inhibit glandular hyperplasia, and promote fibrous tissue absorption. They can adjust the liver’s ability to inactivate hormones, improve liver and systemic blood circulation, and promote hormone metabolism in the body.

Herbs for resolving phlegm, softening hardness, and dispersing nodules can promote the absorption of pathological products and inflammatory exudates, promote the dissipation of fibrous tissue and other substances around the glands, thus inhibiting breast gland hyperplasia. The iodine components in herbs for reducing swelling and dissipating nodules can stimulate the production of P in the anterior pituitary gland, reduce estrogen levels in the body, thus can be used to treat breast hyperplasia.

### 4.6. High frequency herbs and herbal pairs

#### 4.6.1. Angelica sinensis

It was recorded in *Jingyue Quanshu·Bencao Zheng: Angelica sinensis, with its sweet and strong fragrance, is specialized in nourishing blood. It is light and pungent, so it can also promote blood circulation. It can nourish blood dynamically, and is known as the holy herb for nourishing blood, promoting blood and Qi circulation.* According to *Medical Enlightenment: Its tail can break blood stasis while its main body can regulate blood.* According to *Compendium of Materia Medica*: *Angelica’s head stops bleeding, the tail flows downwards, its whole body enriches blood and activates blood circulation.* It was also recorded in *Rihuazi Herb*: *Angelica can eliminate damaged blood and promote the generation of fresh blood.*

The main components of *Angelica sinensis* include volatile oil, ferulic acid, vitamin A, vitamin B, vitamin E, and 23 essential inorganic elements for the human body. It can be used to treat blood stasis caused by various pathogenic factors, and can also significantly improve various hemodynamic indicators. Its pharmacological mechanism is as follows:

##### 4.6.1.1. Effects on the hematopoietic system

*Angelica sinensis* is a natural inducer that can not only promote normal hematopoiesis, but also inhibit abnormal cells proliferation, or induce their apoptosis or differentiation.

Angelica polysaccharides can affect the hematopoietic system, and significantly promote the proliferation and differentiation of mouse hematopoietic stem cells (Colony-Forming Unit-Spleen), mouse and human myeloid progenitor cells (Burst-Forming Unit-Erythroid, Colony-Forming Unit-Erythroid, Colony-Forming Unit-Granulocyte/Macrophage, and Colony-Forming Unit-Megakaryocyte).

Bone marrow macrophages induced by *Angelica* polysaccharides can increase the colony formation of myeloid hematopoietic progenitor cells. After induction by *Angelica sinensis*, the levels of erythropoietin, granulocyte-macrophage colony-stimulating factor (GM-CSF), interleukin 3, and interleukin 6 secreted by bone marrow macrophages increased to varying degrees. The mRNA expression levels of erythropoietin and GM-CSF were significantly increased. *Angelica* polysaccharides can also promote the synthesis and secretion of hematopoietic regulatory factors at both the genetic and protein levels, thereby promoting the proliferation and differentiation of myeloid pluripotent hematopoietic progenitor cells, advanced erythroid progenitor cells, and granulocytic progenitor cells.

*Angelica* polysaccharide can also significantly stimulate the proliferation of hematopoietic progenitor cells, the *Angelica* polysaccharide-induced conditioned medium of human thymocytes, glandular cells, and bone marrow stromal cells can significantly promote the proliferation of hematopoietic progenitor cells. Stimulation with *Angelica* polysaccharides in vitro significantly increased the expression levels of GM-CSF protein and mRNA in bone marrow stromal cells, spleen cells, and thymocytes.

*Angelica* polysaccharides can not only increase the number of peripheral white blood cells, red blood cells, hemoglobin (Hb), etc; but also significantly promote the proliferation and differentiation of hematopoietic stem cells and progenitor cells, and improve the hematopoietic function of the body in various aspects.

*Angelica sinensis* can replenish blood by significantly improving the peripheral blood count. It can also reduce spleen index, and increase the liver and thymus indexes. The mechanism of blood tonifying is mainly related to its polysaccharide affecting the hematopoietic system and promoting hematopoiesis.^[33]^

*Angelica* polysaccharides can accelerate DNA synthesis in bone marrow nucleated cells of blood deficient animals, increase the number of bone marrow hematopoietic cells, promote blood cell differentiation and maturation, and restore the peripheral blood cell count in blood deficient animals.^[34]^

*Angelica* polysaccharides can protect and improve the hematopoietic microenvironment, induce and activate fibroblasts, lymphocytes, and macrophages in the hematopoietic microenvironment, and exert hematopoietic effects.^[35]^

It can protect against radiation-induced oxidative damage of bone marrow mononuclear cells, promote the recovery of hematopoietic function and the increase of blood cells, which have obvious protective effects on radiation-induced hematopoietic injury.^[36]^

In addition, it can significantly enhance the expression of VEGFA, enhance the proliferative capacity of bone marrow stem cells, and reduce their apoptosis, which accelerates the recovery of hematopoietic function from acute radiation-induced injury in multiple ways.^[37]^

*Angelica* polysaccharides can also delay or antagonize the hematopoietic function of bone marrow in aging model rats with immune deficiency caused by d-galactose, and have antiaging effects.^[38]^

*Angelica* polysaccharides can not only promote hematopoiesis, but also exert bidirectional regulatory effects in hemostasis through different mechanisms, which can both prevent blood clotting and promote platelet aggregation.^[39]^

##### 4.6.1.2. Effects on the coagulation system

Volatile oil of *Angelica sinensis* can prolong the coagulation time, prothrombin time (PT), and activated partial thromboplastin time by inhibiting platelet aggregation.^[40,41]^ Additionally, it can also significantly decrease WBV, whole blood reductive viscosity and hematocrit.^[42]^

Ferulic acid can significantly increase the white blood cell and red blood cell counts, and levels of Hb, iron, zinc, and copper in blood, simultaneously reduce hematocrit and whole blood low shear viscosity.^[43]^

An *Angelica sinensis* Decoction can reduce plasma viscosity, improve coagulation function and histopathological damage in rats with acute blood stasis, the effects of complete *Angelica* and *Angelica* tail are more significant than those of *Angelic*a body.^[44]^

*Angelica sinensis* can reduce the red blood cell deformation index and fibrinogen content in MGH patients (*Qi* stagnation and blood stasis), and improve the patients’ blood rheology. It can also reverse the formation of blood stasis by regulating amino acid and lipid metabolism in rats with blood stasis syndrome.^[45]^

##### 4.6.1.3. Antiplatelet aggregation and anti-thrombosis effects

*Angelica sinensis* injection can improve microcirculation disorders, reduce platelet aggregation, and increase organ blood perfusion in hemorrhagic shock rats.^[46]^

The volatile oil of *Angelica sinensis* has also shown significant antagonistic effects on adenosine diphosphate (ADP)-induced platelet aggregation.^[40]^

The total phthalides in *Angelica* volatile oil can significantly prolong arterial thrombosis time, improve the viscosity of whole blood and plasma in hyperviscoemia rats. By inhibiting platelet aggregation, it can affect the coagulation system and lengthen indicators, such as PT, activated partial thromboplastin time, thrombin time (TT).^[47]^

The *Angelica* volatile oil components (*N*-butylphenol and ligustilide) have inhibitory effects on platelet aggregation. In terms of inhibiting platelet aggregation, *Angelica* volatile oil is stronger than nonvolatile ingredients, while the activity of nonvolatile components was stronger than that of volatile oils in terms of the PT.^[48]^

The ferulic acid in *Angelica* has significant effects on dilating coronary vessels, increasing coronary flow, improving myocardial ischemia, and anti-thrombogenesis.^[34]^

Ferulic acid can increase the number of bone marrow nucleated cells, improve blood rheology, enhance red blood cell deformability, and inhibit platelet aggregation and adhesion.

Ferulic acid derivatives can also significantly inhibit ADP-induced platelet aggregation. Sodium ferulate can significantly increase the ratio of prostacyclin I_2_ (PGI_2_) to thromboxane A_2_ (TXA_2_), inhibit platelet aggregation and the release of thromboxane-like substances, and selectively inhibit the activity of TXA_2_ synthetase.^[49]^

##### 4.6.1.4. Anti-inflammatory and analgesic effects

*Angelica* volatile oil can inhibit edema and exudation in the early stage of inflammation, as well as tissue proliferation and granulation tissue formation in the late stage of inflammation. In addition, it has inhibitory effects on acetic acid-induced mouse torsion reaction.^[50]^

The A3 active site of *Angelica* can dose-dependently inhibit xylene-induced ear swelling and carrageenan-induced toe swelling, and its mechanism is related to the inhibition of cyclooxygenase-2 mRNA and protein expression.^[51]^

#### 4.6.2. Spatholobus Suberect Stem

It is recorded in *Modern Practical Herbs*: *Spatholobus Suberect* Stem tastes bitter and slightly sweet, has a mild nature, belongs to the heart and spleen meridians, and has the effects of promoting blood circulation, relaxing tendons, nourishing blood and regulating menstruation. There have been many discussions on it in ancient medical works: *Greatly tonifying Qi and blood is more effective for the elderly and women* (*A Supplement to Compendium of Materia Medica*). *Spatholobus Suberect Stem can remove blood stasis, generate new blood, and smooth meridians* (*Yinpian Xincan*). It is a powerful blood tonic, suitable for anemia-induced nerve paralysis and gynecological diseases, with the effects of promoting blood circulation and relieving pain.

The main components of *Spatholobus Suberect* Stem include *Spatholobus Suberect* Stem alcohol, iron, rapeseed sterol, stigmasterol, and sitosterol. It promotes hematopoiesis by increasing red blood cells and Hb, and plays a role in nourishing blood. It can also prevent platelet aggregation, increase arterial blood flow, and reduce vascular resistance.

#### 4.6.3. Salvia miltiorrhizae

*Salvia miltiorrhizae* is the dry root and rhizome of *Salvia miltiorrhiza* Bge. in the Lamiaceae family. It tastes bitter and slightly cold, belongs to the heart and liver meridian. It has the effects of activating blood circulation, removing blood stasis, relieving pain through menstruation, clearing the heart’ heat and eliminating irritability, cooling blood and eliminating carbuncle.

Many ancient medical works have records of *Salvia miltiorrhiza*. It was recorded in *Materia Medica Biandu*: *Salvia miltiorrhizae has the same effect as the Siwu Decoction, it can dispel blood stasis to generate new blood, is good at dispelling wind and removing nodules. It has a peaceful nature to treat blood diseases, and has sweet and bitter taste to regulate meridians. Salvia miltiorrhizae can nourish blood, calm nerves, dredge arthrosis, relieve swelling and pain, promote muscle growth, break blood stasis and replenish new blood, stop bleeding, treat pain and discomfort of the limbs, regulate women’s menstruation, and treat anxiety and scabies* (*Rihuazi Herbal*). *Downward movement is the characteristic of Salvia miltiorrhizae that can promote blood circulation and is suitable for those with blood heat and stagnation. Therefore, it is an important herb for gynecological menstrual regulation and postpartum treatment* (*Chongqingtang Suibi*).

The main components of *Salvia miltiorrhizae* include tanshinone, cryptotanshinone, protocatechualdehyde, protocatechuic acid, tanshinol, vitamin E flavonoids, triterpenes, sterols, etc. *Salvia miltiorrhizae* can protect against platelet aggregation, and thrombosis, dilate coronary arteries and peripheral blood vessels, promote fibrinolysis, which have anticoagulant effects.

#### 4.6.4. Astragalus mongholicus

*Astragalus mongholicus* is the root of *Leguminous mongholicus.* It has sweet taste and slightly warm nature, belongs to the spleen and lung meridians. It has the effects of strengthening spleen, nourishing the whole body, lifting positive energy, expelling toxins and generating muscles, and is used to treat diseases induced by deficiency of *Qi* and blood.

The main manifestations of *Qi* deficiency in TCM theory are structure damage and production capacity changes in mitochondria. Breast hyperplasia is a disease characterized by evil excess and positive energy deficiency. *Astragalus mongholicus* has a therapeutic role in treating breast hyperplasia by nourishing vital energy. Its main mechanisms of action are as follows.

First, it can ameliorate mitochondrial outer membrane damage, as well as the activity of intimal membrane potential, cytochrome C oxidase and monoamine oxidase, protect their structure and function, and regulate energy metabolism.^[52]^

Second, when the body’s positive energy is deficient, the body’s immune function is impaired. It is also the main type of MGH. *Astragalus* can significantly change the proportion of T cells, B cells, and CD8 (Cluster of Differentiation 8) T cells to natural killer cells, as well as the expression levels of C-X-C motif chemokine receptor 3, differentiation antigen cluster CD69 molecule and selectin L.^[53]^

Third, *Astragalus* can exert its effects of tonifying *Qi* and promoting blood circulation by regulating the immune system. It can also reduce WBV, hematocrit, and aggregation at various shear rates, enhance the deformability of red blood cells, and improve their function. Additionally, it has a positive effect on improving hemodynamics and hemorheology.^[54]^

#### 4.6.5. Safflower

Safflower is the dry tubular flower of the Asteraceae plant (*Carthamus tinctorius,* common name Safflower), with acrid taste and mild nature. It belongs to the heart and liver meridians. It has the effects of promoting blood circulation, dredging meridians and collaterals, removing blood stasis and relieving pain, and is used to treat amenorrhea, fistula, dystocia, swelling, pain caused by blood stasis, and injuries caused by falls. It was recorded in *Herb Yanyi Buyi*: *A large dose of Safflower is used to break blood stasis, while less is used to nourish blood*.

The main components of Safflower are flavin, glycoside, Safflower pigment, quinone glycoside, neosafflower glycoside, Safflower oil chlorogenic acid, caffeic acid, catechol, pyrocatechol, etc. Safflower can dilate arteries, prevent coagulation and thrombus, increase coronary blood flow and myocardial nutrient blood flow, inhibit platelet aggregation, enhance fibrinolysis, and reduce WBV.

Safflower flavin has inhibitory effects on ADP-induced platelet aggregation, as well as deaggregation on ADP-aggregated platelets. Safflower flavin can significantly prolong plasma calcium recovery time, PT and coagulation time. It also affects the coagulation system in vivo and in vitro, and has very significant inhibitory effects on thrombus, which can improve microcirculation, protect against inflammation and platelet aggregation, as well as effectively enhance the body’s cellular immunity, humoral immunity and nonspecific immune function. Safflower oil also has the effect of reducing blood lipids.^[55]^

#### 4.6.6. Peach Pit

*Peach* (*Prunus persica* (L.) Batsch, *Prunus davidiana* (Carr.) Franch) *Pits* are dry and mature seeds of plants in the Rosaceae family, which has a bitter and sweet taste, and a peaceful nature. It belongs to heart, liver, and large intestine meridians.

*Peach Pit* is an important herb for promoting blood circulation and resolving stasis, and can also moisten intestines and relieve constipation. Pharmacological studies have shown that the water extract of *Peach Pit* can effectively inhibit platelet aggregation and inflammatory responses, and significantly improve the body’s hemodynamics.

#### 4.6.7. Ligusticum wallichii

*Ligusticum wallichii* is the rhizome of the umbrella plant (*Ligusticum striatum*, *Ligusticum wallichii*) that is used as a herb. It has a spicy taste and is mild in nature. It has the effects of dispelling wind, relieving pain, promoting blood and *Qi* circulation.

Pharmacological studies have shown that the compound ligustrazine present in *Ligusticum wallichii* can effectively improve microcirculation and hemorheology, as well as protect against platelet aggregation and thrombosis.^[56]^

### 4.7. Herbal pairs

#### 4.7.1. *Astragalus mongholicus + Angelica sinensis*

*Astragalus mongholicus* is good at strengthening the function of spleen, nourishing *Qi* and blood, while *Angelica sinensis* can replenish blood and promote blood circulation. The principle of combining the 2 is to simultaneously regulate *Qi* and blood, so as to play the role of supplementing *Qi*, nourishing blood, and promoting blood circulation.

*Qi* deficiency and blood stasis generally lead to breast hyperplasia. *Wang Qingren* (*Qing* Dynasty) explicitly proposed in his book (*Yilin Correcting Mistakes*) the following: *If the vitality (fundamental Qi) is deficient, it must not reach the blood vessels, the blood vessels lack the nourishment of Qi, and the blood must not run normally and be stagnant. Qi* deficiency is mainly manifested as reduced visceral function or decreased immunity; blood stasis refers to the accumulation of blood in the meridians or organs due to poor blood flow or blood deviation from the meridians, and is mainly manifested as abnormalities in hemorheology and hemodynamics, accompanied by dysfunction of vascular endothelial function.^[57]^

##### 4.7.1.1. Regulation of energy metabolism

Mitochondria are important sites for energy generation and metabolism. The herbal pair (*Astragalus mongholicus* + *Angelica sinensis*) can regulate mitochondrial bioenergy generation, increase membrane potential, upregulate mitochondrial gene expression, regulate autophagy, and improve mitochondrial damage.^[58,59]^

The mitochondrial function impairment in H9c2 cells was repaired by promoting BCL2 expression, increasing the release of cytochrome *c* and reducing the production of reactive oxygen species (ROS). Cyclic guanosine monophosphate and cyclic adenosine monophosphate (cAMP) are important regulatory signaling compounds in various cellular processes that play important roles in energy metabolism, and are often associated with the “*Yin-Yang*” in TCM theory. The herbal pair (*Astragalus mongholicus* + *Angelica sinensis*) can significantly increase the cAMP/cyclic guanosine monophosphate value in myocardium, and their nourishing effect on *Qi* are associated with increased cAMP levels in the body.

This herbal pair can promote aerobic glycolysis, regulate energy metabolism, and enhance physical fitness by reducing serum lactate and lactate dehydrogenase levels.^[60]^

##### 4.7.1.2. Antioxidant stress

Oxidative stress manifests as an increase of intracellular ROS and other free radicals, which leads to an imbalance between oxidation and antioxidation, directly or indirectly causing oxidative damage to cells or tissues. The herbal pair (*Astragalus mongholicus* + *Angelica sinensis*) can increase the serum levels of SOD and glutathione peroxidase 1, reduce the content of MDA, reduce serum levels of nitric oxide (NO), ROS, and 8-isoprostaglandin F2α, as well as increase the level of heme oxygenase 1.^[61]^

##### 4.7.1.3. Anti-inflammatory effects

The role of the herbal pair (*Astragalus mongholicus* + *Angelica sinensis*) in nourishing *Qi* and promoting blood circulation is related to their anti-inflammatory effects. *Qi* deficiency and blood stasis can both lead to inflammatory reactions, which mainly manifest by affecting the expression of inflammatory cytokines and chemokines, and regulating the signaling pathways related to inflammatory responses. At the same time, oxidative stress can also cause endogenous damage to the body, activate the release of inflammatory mediators, trigger inflammatory responses, as well as affect the function of the immune system, blood circulation, and physiological functions of organs and tissues. The herbal pair (*Astragalus mongholicus* + *Angelica sinensis*) can reduce the expression of inflammatory related factors, such as tissue necrosis factor (TNF), intercellular adhesion molecule 1, interleukin-lA, interleukin 4, and interleukin 6. This herbal pair regulates the balance of T helper 1 cells/T helper 2 cells (Th2) cytokines and has anti-inflammatory effects by inhibiting signal transducer and activator of transcription 6, inducing the expression of signal transducer and activator of transcription 4, and regulating Th2, specific transcription factor T-box transcription factor 21, and GATA (GATA-binding protein transcription factor) binding protein 3. The above inflammation and oxidative stress processes are both induced by weak *Qi* and blood, but the above effects of this herbal pair are also reflected in their effects of nourishing *Qi* and promoting blood circulation.^[62,63]^

##### 4.7.1.4. Regulating immune function

*Qi* deficiency and blood stasis are often accompanied by decreased and imbalanced immune function, while the herbal pair (*Astragalus mongholicus* + *Angelica sinensis*) can regulate immune function by regulating T cells and the expression of cytokine genes, which play a beneficial role in nourishing *Qi*.

This herbal pair can promote the production of cyclophosphamide-induced serum immunoglobulin M and improve the lymphocyte transformation rate.^[64]^

The herbal pair (*Astragalus mongholicus* + *Angelica sinensis*) can significantly increase the spleen index of rats with *Qi* deficiency and blood stasis, significantly decrease the expression of interferon gamma (IFNG), and an increase of the interleukin 4. By regulating the T helper 1 cells/Th2 balance, this herbal pair plays a role in enhancing immunity and regulating immune stability.^[65]^

This herbal pair also interferes with the activation of the eukaryotic translation initiation factor 2 signaling pathway by influencing the IFNG-induced T-box transcription factor 21 expression in bone marrow cells in the bone marrow microenvironment, thereby restoring the balance of the T cell immune response network.^[66]^

This herbal pair can regulate the number of T cell subpopulations, transformation and proliferation capacity, adrenal function of rats with *Qi* deficiency and blood stasis, downregulate the mRNA expression of TNF, interleukin 1B, nuclear transcription factor kB, mitogen-activated protein kinase 14, and mitogen-activated protein kinase 8 by regulating the nuclear transcription factor kB, p38 mitogen-activated protein kinase and c-Jun N-terminal kinase immune response signaling pathways.^[67]^

The complement C3b/C4b receptor 1 (CR1) on the red cell membrane, the C3b molecule in the attached immune complex or the C3b molecule in the antigenic complement complex can respectively adhere to yeast polysaccharides, complement-sensitized yeast and -non-sensitized yeast, to form rose wreaths (E-C3bRR) and immune complex wreaths (E-ICR). The herbal pair (*Astragalus mongholicus* + *Angelica sinensis*) can strengthen the adhesion function of red cells by enhancing E-C3bRR/ E-ICR (Erythrocyte-C3b Receptor/ Erythrocyte-Immune Complex Receptor), and then improve the immune level.^[68]^

##### 4.7.1.5. Impact on the blood system

Blood stasis often manifests as a state of viscosity, high concentration, condensation and aggregation, as well as changes in tangible composition (increased blood lipids and blood sugar). The herbal pair (*Astragalus mongholicus* + *Angelica sinensis*) can reduce blood viscosity, blood lipids, and blood sugar levels. Besides its blood activating effect, it is also associated with the regulation of substance metabolism, which reflects its *Qi* tonifying effect.

This herbal pair (*Astragalus mongholicus* + *Angelica sinensis*) can significantly reduce the whole blood low shear viscosity in rats with *Qi* deficiency and blood stasis.^[65]^ It can also increase the ratio of 6-ketoprostaglandin-F_1α_ to thromboxane-B_2_, regulate platelet aggregation, enhance the fluidity and deformability of the red blood cell membrane, reduce the hematocrit of whole blood red cells, reduce the plasma NO content, inhibit the overexpression of integrin subunit beta 2, and intercellular adhesion molecule 1, reduce the adhesion rate of white blood cells and whole blood specific viscosity.^[69]^

##### 4.7.1.6. Intervention in angiogenesis

Blood stasis is a pathological condition caused by the break of the balance between blood fluid and blood tube, which results from either restrained or overactive circulation of blood fluid.^[70]^ This herbal pair can have the effect of tonifying *Qi* and promoting blood circulation by bidirectional regulation of angiogenesis, which maintains the stability and balance of the blood system. Endothelial progenitor cells (EPCs) are precursor cells of vascular endothelial cells, which participate in the formation of new blood vessels after vascular injury. This herbal pair can maintain the function of EPCs by upregulating the levels of NO/nitric oxide synthase 3/AKT1, can increase the autophagy level of bone marrow EPCs in mice injured by oxidative stress, and promote their migration and proliferation capacity. Additionally, through activation of the PI3K/Akt/MAPK signaling pathway, it promotes the VEGFA expression in ischemic regions, and promotes angiogenesis.^[71]^

Moreover, in rats with liver fibrosis, it can reduce VEGFA, angiotensin I, and transforming growth factor beta 1, as well as the expression of its signal transmission medium, exert antiangiogenic effects by alleviating oxidative stress.

#### 4.7.2. *Angelica sinensis *+ *Spatholobus Suberect Stem*

The iron in the herbal pair (*Angelica sinensis* + *Spatholobus Suberect Stem*) has significant effects on promoting the generation of red blood cells, increasing Hb contents, and promoting bone marrow hematopoietic function, which reflect the blood nourishing effects of the 2 herbs. In addition, the herbal pair can expand coronary blood flow, prevent platelet aggregation, enhance fibrinolysis, which play the role of blood activation.^[72–74]^

This herbal pair can also inhibit the expression of liver ferritin, increase the accumulation of endothelial PAS domain-containing protein 1 protein, restore erythropoietin synthesis, activate the Janus kinase 2/signal transducer and activator of transcription and PI3K/Akt signaling pathways. It can restrain the overexpression of elemental iron regulatory factors by regulating the hepcidin coding gene (hepcidin antimicrobial peptide), which improve iron metabolism, and can be used to treat breast hyperplasia induced by blood deficiency.

The herbal pair (*Angelica sinensis* + *Spatholobus Suberect Stem*) performs molecular functions, such as facilitating the binding of identical proteins, cytokine active enzymes, protein phosphatases, and transcription factors through several biological processes, such as positive regulation of gene expression, positive regulation of RNA polymerase II promoter transcription, negative regulation of extracellular space-mediated cell apoptosis, cytosol, cytoplasmic membrane, thus it can be used to treat breast hyperplasia with blood deficiency.

The herbal pair (*Angelica sinensis* + *Spatholobus Suberect Stem*) promotes the generation of red blood cells, regulates the inflammatory state of the body, improves iron utilization by activating multiple pathways through multiple components and targets, thus it plays a role in treating breast hyperplasia with blood deficiency. The HIF-1 [(Hypoxia-Inducible Factor-1 Alpha Subunit, HIF1A), (Hypoxia-Inducible Factor-1 Beta Subunit, HIF1B)] signaling pathway is directly involved in hematopoiesis, and Hypoxia-Inducible Factor-1 (HIF-1, HIF1A and HIF1B) is a transcription factor crucial in maintaining oxygen levels in the body, by regulating systemic oxygen delivery through Hb. The HIF regulatory subunit HIF1A can promote the production of endogenous erythropoietin in the kidneys, and HIF1A can increase iron absorption, accessibility of iron storage, and iron transport, as well as reduce the expression of iron regulatory factors, thereby improve the inflammatory response and increasing iron utilization in the body.^[75]^

The TNF signaling pathway is involved in the inflammatory response. The herbal pair (*Angelica sinensis* + *Spatholobus Suberect Stem*) alleviates inflammation by regulating the TNF signaling pathway, and maintains red blood cells’ homeostasis by inducing stress erythropoiesis.^[76]^

#### 4.7.3. *Sedge + Aromatic Turmeric Root-tuber*

*Sedge* + *Aromatic Turmeric Root-tuber* is a classic herbal pair for soothing the liver and promoting blood circulation. *Sedge*, which has a pungent, slightly bitter, slightly sweet and mild in nature taste, belongs to liver and spleen meridians, and has the effects of promoting *Qi* circulation, relieving depression and pain. *Aromatic Turmeric Root-tuber*, which has a pungent and bitter taste, and is cold in nature, belongs to liver, heart and lung meridians. It has the effects of promoting blood circulation to relieve pain, promoting *Qi* circulation to relieve depression, clearing heart fire, cooling blood, and is mainly used for treating *Qi* stagnation and blood stasis pain. *Aromatic Turmeric Root-tuber* can smooth the twelve meridians patency, promote the movement of *Qi* in the blood, and is very effective for regulating *Qi* and resolving depression. However, its function of promoting blood circulation is insufficient, and more significant therapeutic effects can be obtained by the aid of *Aromatic Turmeric Root-tuber* (activating blood and removing stasis). *Sedge can* promote *Qi* circulation to promote blood circulation, while *Aromatic Turmeric Root-tuber* can promote blood circulation to promote *Qi* circulation. The 2 are used together and complement each other, which play a role in soothing the liver, relieving depression, promoting blood circulation and regulating *Qi*.

The effects of the main effective components of the herbal pair (*Sedge* + *Aromatic Turmeric Root-tuber*) are as follows^[77–79]^:

Sitosterol and stigmasterol can prevent arrest of breast cells in the DNA synthesis phase of cell division, inhibit cell proliferation, and promote cell apoptosis.

Phytosterols can enhance the proliferation of peripheral blood lymphocytes and T cells, enhance the immune response, and prevent the development of MGH from the perspective of immunosuppression.^[80]^

Isorhamnetin can promote cell apoptosis by binding with autophagy/mitosis inhibitors, and can also inhibit breast cell proliferation by inhibiting cell growth, causing cytotoxicity, increasing oxidative stress, and blocking cell cycle progression.^[81,82]^

Kaempferol is associated with multiple signaling pathways, such as the EGFR (Epidermal Growth Factor Receptor)/PI3K/Akt, and Notch1 (Neurogenic locus notch homolog protein 1)/PI3K/Akst pathways, upregulates proapoptotic proteins BAX and CASP3, downregulates antiapoptotic protein BCL2, thereby blocking cell cycle and promoting cell apoptosis.^[83]^

Kaempferol can also significantly reduce the expression of interleukin 6 and interleukin 1B, and exhibits a dose dependence, thereby alleviating the degree of breast hyperplasia. Quercetin is a natural plant hormone with estrogenic activity, similar in structure to E_2_. It can bind to the ERs, regulate hormone levels, induce cell apoptosis by downregulating antiapoptotic factor BCL2 and upregulating proapoptotic factors BAX and BAK1 (BCL2 antagonist/killer 1).^[84–86]^

### 4.8. Core herbal combinations and herbal pairs originating from ancient prescriptions

#### 4.8.1. Xuefu Zhuyu Decoction

The *Xuefu Zhuyu Decoction* originated from the ancient medical book *Medical Forest Correct Mistakes*, which is a prescription for regulating blood in TCM. Among its effects are the following: promoting blood circulation, removing blood stasis, relieving pain, and promoting *Qi* circulation, and is used to treat blood stasis in the chest, whose main symptoms include chest pain, headache, pain like acupuncture fixed and persistent, palpitations, insomnia and dreaminess, irritability, dark lips and eyes, dark lips and eyes, dark red or ecchymosis tongue, and astringent or tight pulse.

The results of this study indicated that many core combinations and herbal pairs originated from the *Xuefu Zhuyu Decoction* (Table 5; Peach Pit + Safflower + *Angelica sinensis* + *Ligusticum wallichii* + Radix paeoniae rubra, Peach Pit + Safflower + *Bupleuri Radix* + Immature Trifoliate orange Fruit, *Angelica sinensis* + Raw rehmannia root + *Ligusticum wallichii* + Radix paeoniae rubra, Peach Pit + Safflower + Platycodon grandiflorum + Radix paeoniae rubra, *Angelica sinensis* + Raw rehmannia root + *Ligusticum wallichii* + Radix paeoniae rubra, *Ligusticum wallichii* + Radix achyranthis bidentatae + Radix paeoniae rubra, *Bupleuri Radix* + Immature Trifoliate orange Fruit + *Glycyrrhiza uralensis* Fisch, *Bupleuri Radix* + Immature Trifoliate orange Fruit + Platycodon grandiflorum). The pharmacological mechanism of this compound in preventing and treating breast hyperplasia is as follows.

##### 4.8.1.1. Improve blood rheology

This prescription can significantly reduce WBV and plasma viscosity, significantly prolong blood clotting time, prevent platelet aggregation, accelerate red blood cell electrophoresis, inhibit thrombosis, reduce TXA_2_ content, increase PGI_2_ level, and significantly reduce TXA_2_/PGI_2_. It can also effectively improve hemorheological indicators and clinical symptoms of high blood viscosity patients.^[87]^

##### 4.8.1.2. Improving microcirculation

The *Xuefu Zhuyu Decoction* can significantly dilate microvessels, improve microcirculation, block the pathological process of microcirculation disorders and promote their recovery, accelerate blood flow rate, increase capillary openings and tissue perfusion, inhibit ADP, induce platelet aggregation, promote platelet depolymerization, enhance reticuloendothelial cell system function, timely removal of clotting substances and fibrin degradation products, thereby reducing the factors triggering blood stasis.

##### 4.8.1.3. Regulation of disorders of lipid metabolism

This prescription can reduce serum low-density lipoprotein (LDL) and cholesterol, reduce LDL synthesis in the liver, increase lipoprotein catabolism, decrease blood lipoprotein, thereby obstructing the transport of total cholesterol and triglycerides from the liver to extrahepatic tissues, leading to a decrease of thereby obstructing the transport of total cholesterol and triglycerides in the blood. It can also indirectly affect cholesterol transport by LDL, and the lipid exchange rate between very low-density lipoprotein and high-density lipoprotein, thus increasing high-density lipoprotein levels in the blood.

##### 4.8.1.4. Anti-anoxic effect

This drug can reduce the overall oxygen consumption by the animal, improve hypoxia tolerance, reduce the oxygen consumption by brain tissue, and prolong the survival time of acute hypoxic animals.^[88]^ It can significantly inhibit the hypoxia-promoting effect on the proliferation of pulmonary artery smooth muscle cells in a dose-dependent manner.^[89]^

#### 4.8.2. Bupleurum Liver soothing Powder

*Bupleurum Liver soothing Powder* is a representative prescription for regulating liver and spleen, derived from the ancient medical works *Jingyue Quanshu*. It is composed of several herbs, including *Orange peel*, *Bupleuri Radix, Ligusticum wallichii, Immature Trifoliate orange Fruit, Chinese herbaceous peony, Glycyrrhiza uralensis* Fisch, and *Sedge*.

In this prescription, *Bupleuri Radix* and *Chinese herbaceous peony* have the effect of soothing the liver and relieving depression, and are the most important components. *Sedge, Immature Trifoliate orange Fruit*, *Unripe orange,* and *Orange peel* can regulate *Qi* circulation, and are important secondary components. *Ligusticum wallichii, Peach Pit, Safflower, Myrrh, Frankincense* serve as supplemental herbs for promoting *Qi* and blood circulation. *Prepared rehmannia root* (nourishing *Yin* and tonifying blood) and *Angelica sinensis* (nourishing blood and promoting blood circulation) are also supplemental herbs.

*Glycyrrhiza uralensis* Fisch can relieve pain and regulate other herbs. The combination of various herbs can soothe liver, regulate *Qi* circulation, disperse nodules, and eliminate swelling. The results of this study indicated that many core combinations and herbal pairs originated from the *Xuefu Zhuyu Decoction* (Table 5; *Bupleuri Radix* + Orange peel + Sedge + *Ligusticum wallichii* + Radix paeoniae rubra, Orange peel + *Ligusticum wallichii* + *Paeonia lactiflora* Pall + Radix paeoniae rubra, Immature Trifoliate orange Fruit + Radix paeoniae rubra + Sedge, *Bupleuri Rad*ix + Immature Trifoliate orange Fruit + *Ligusticum wallichii* grandiflorum, *Bupleuri Radix* + Immature Trifoliate orange Fruit + *Glycyrrhiza uralensis* Fisch).

In mammary gland, as a target organ for liver *Qi* release, liver *Qi* stagnation causes the environmental disorder of the mammary gland, as well as an imbalance between proliferation and regression of mammary ductal epithelial cells, which lead to dysfunction of the mammary ductal microecology. In addition, liver *Qi* stagnation leads to liver dysfunction, *Qi* stagnation and blood stasis, and disorder of *Chong* and *Ren* meridians. *Qi* and blood block in breast meridians, leads to the formation of breast lumps, and results in breast hyperplasia.

*Bupleurum Liver soothing Powder* can exert antidepressant effects through multiple pathways and targets, and can prevent MGH by interfering with the release of hormones (such as FSH, E_2_, 5-hydroxyserotonin (5-HT), PRL, VEGFA, LH, etc). It can regulate the body’s endocrine system.^[90]^

The regulatory effects of *Bupleurum Liver soothing Powder* on the endocrine system is manifested in the regulation of the hypothalamic-pituitary-ovarian and hypothalamic–pituitary–adrenal axes. It alleviates nipple discharge, breast pain and lumps, reduces breast gland thickness, and breast duct diameter by reducing prolactin and E_2_ levels, thus increasing P levels.^[91]^

Among the components of the prescription, *Paeoniflorin* can improve local blood circulation, regulate hormone levels, and achieve dynamic balance^[92]^; *Angelica sinensis* volatile oil acts like P on the uterus, counteracts elevated levels of E_2_, and regulates disordered hormone^[93]^; Sedge volatile oil has antidepressant, analgesic, estrogenic, and tumor cell apoptosis-inducing effects^[94]^; Ligustrazine has the effects of preventing platelet aggregation, inhibiting thrombosis and relieving pain.^[95]^

#### 4.8.3. Siwu Decoction

The *Siwu Decoction* is a basic composition for nourishing blood and regulating menstruation derived from the ancient medical book *Taiping Huimin Heji Bureau Prescription*, which has the effects of nourishing blood, regulating menstruation and resolving blood stasis. *Angelica sinensis* is the main herb for tonifying blood, nourishing liver, regulating blood circulation and menstruation; *Rehmannia glutinosa* is the main secondary herb for nourishing *Yin*-essence and blood, *Paeonia lactiflora Pall* (nourishing blood, softening liver, regulating blood) and *Ligusticum wallichii* (promoting the circulation of blood and *Qi*, unblocking *Qi* and blood flow) are supplemental herbs. *Rehmannia glutinosa* and *Paeonia lactiflora Pall* nourish *Yin*-essence and blood, while *Angelica sinensis* and *Ligusticum wallichii* promote *Qi* and blood circulation. The 4 herbs are compatible with each other to achieve a combination of movement and stillness, which replenish blood without leaving stasis, and promote blood circulation without damaging blood. It is a commonly used prescription for treating blood deficiency and poor blood circulation. After the *Song* Dynasty, *Paeonia lactiflora Pall* and *Radix paeoniae rubra* were discussed separately, so *Radix paeoniae rubra* was added to the *Siwu Decoction*.

The results of this study indicated that the core combination originated from the *Siwu Decoction* (Fig. 5; *Angelica sinensis* + *Raw rehmannia* root + *Ligusticum wallichii* + *Radix paeoniae rubra* + *Paeonia lactiflora Pall*). The pharmacological mechanism of its treatment of breast hyperplasia with blood deficiency is as follows:

The *Siwu Decoction* can improve the peripheral blood picture of anemic mice, and increase the expression of transferrin receptor/Ly antigen 76, a surface marker antigen on peripheral blood and spleen red blood cells.^[96]^ Its mechanism of improving anemia is related to its ability to regulate iron metabolism by improving ferritin.^[97]^ The above process is related to its protection of liver cells.

The *Siwu Decoction* can reduce liver cell apoptosis by inhibiting the activation of CASP3 and caspase 8, reducing the expression of the *BAX* and *FAS* genes. It upregulates the expression of BCL2 and BCL2L1 proteins in bone marrow cells, reduces the apoptosis rate of bone marrow cells, improves peripheral hemogram, and thus plays a role in nourishing blood.^[98]^

The *Siwu Decoction* promotes the entry of bone marrow cells into the cell cycle, specifically enhances hematopoietic function and mediates bone marrow generation, by increasing the secretion of interleukin 3, and inhibiting the secretion of IFNG.^[99]^

The above research reveals the objectivity of TCM theory (kidney storing essence, bone generating marrow, and marrow generating blood) from the perspective of modern medicine.^[100]^

#### 4.8.4. Xiaoyao Powder

*Xiaoyao Powder* is derived from the ancient medical works *Taiping Huimin He Ji Ju Fang* and consists of 8 herbs: *Bupleuri Radix, Angelica sinensis, Largehead Atractylodes Rhizome, Paeonia lactiflora Pall, Poria cocos, Glycyrrhiza uralensis* Fisch*, Mint* and *Ginger*, which have the effects of soothing the liver, strengthening spleen, nourishing blood, and regulating menstruation. It is used to treat some symptoms caused by liver depression and spleen deficiency, such as depression, chest discomfort, hypochondriac pain, dizziness, decreased appetite, menstrual irregularities, etc. *Xiaoyao Powder* is often used in the clinical treatment of gynecological diseases, immune diseases, liver diseases, stress injury, endocrine diseases, neurological diseases, etc.^[101]^

The results of this study indicated that some core combinations originated from the *Siwu Decoction* (Fig. 5; *Bupleuri Radix* + *Angelica sinensis* + Paeonia lactiflora Pall + Largehead Atractylodes Rhizome + Poria cocos, Largehead Atractylodes Rhizome + Poria cocos + Paeonia lactiflora Pall + *Glycyrrhiza uralensis* Fisch, *Angelica sinensis* + Largehead Atractylodes Rhizome + Radix paeoniae rubra + Poria cocos, *Bupleuri Radix* + *Angelica sinensis* + Radix paeoniae rubra, *Bupleuri Radix* + Poria cocos + Largehead Atractylodes Rhizome).

*Bupleuri Radix* can sooth the liver and alleviate depression, *Angelica sinensis* can nourish blood and regulate *Qi*, while *Paeonia lactiflora Pall* can nourish blood and *Yin*-essence, and relax the liver. The above 3 herbs are used together to tonify and regulate the liver. Blood filling plays a role in softening and nourishing the liver, and normal blood physiological function can maintain normal liver function.

The combination of *Largehead Atractylodes Rhizome*, *Poria cocos*, and *Glycyrrhiza uralensis* Fisch not only strengthens spleen, but also benefits *Qi*, so that the *Yin-essence* and blood can be produced continuously. Modern pharmacological research has indicated that *Xiaoyao Powder* can increase E_2_ level in female mice, but in breast hyperplasia caused by excessive E_2_, it can also reduce serum E_2_ level and exert an antagonistic effect on E_2_.^[102]^

*Xiaoyao Powder* can reduce the breast diameter and nipple height of rabbits with breast hyperplasia, reduce glandular secretions and ductal epithelial hyperplasia. It can also play a role in anti-inflammatory effects, analgesic effects, and microcirculatory improvement, making the structure of breast tissue clearer, reducing its density, and reducing or dissipating nodules.

### 4.9. Clinical application and development of drugs from high frequency herbs (herbal pairs and combinations)

#### 4.9.1. Optimization of clinical medication

High frequency herbs and core herb combinations can provide the evidence-based foundation for herbal selection in the clinical treatment of MGH. Based on their high frequency efficacy, standardized prescriptions can be formulated to improve the consistency and reproducibility of therapeutic effects.

#### 4.9.2. Guidance for personalized treatment plans

Precise compatibility can be achieved by analyzing the constitution and syndrome types of patients (such as *Qi* stagnation and blood stasis, liver depression and spleen deficiency), and combining with the synergistic effects of high frequency herbal pairs (such as promoting blood circulation + regulating *Qi*). For example, for patients with liver depression, the herbal combination of *Bupleuri Radix, Immature Trifoliate orange Fruit* and *Unripe orange* is preferably selected, while for patients with blood stasis, the combination of Peach Pit, Safflower and *Angelica sinensis* is selected.

#### 4.9.3. Symptom-based adjuvant treatment

High frequency herbs with the effects of clearing heat and detoxifying (such as taraxacum, and honeysuckle) and softening hardness and dissipating nodules (such as *Prunella vulgaris* L, and Oyster) can be used to relieve accompanying symptoms such as breast pain and nodules, which is used to enhance clinical treatment methods.

#### 4.9.4. Guidance for the development of new compound drugs

Core combinations can serve as the basis for the research and development of new drugs. For example, based on the combination of “promoting blood circulation and removing blood stasis + soothing liver and regulating *Qi*” (*Salvia miltiorrhizae*, Bupleuri Radix, and Sedge), a standardized basic formula can be developed. By combining with modern processes to optimize the extraction methods, the stability and bioavailability of active ingredients (such as tanshinone or ferulic acid) can be ensured.

#### 4.9.5. Facilitation of quality standardization and formulation innovation of herbal prescriptions

Based on this study, the herbal fingerprint spectrum and quality control standards for active ingredients (such as *Angelica polysaccharides* or *Astragalus saponins* etc) can be established. In combination with nano-drug delivery and sustained-release technologies, the drug delivery efficiency can be improved and the adverse reactions can be reduced.

The high frequency herbs and their combinations provide a scientific basis for the MGH treatment. In the future, the standardization and modernization of compound drugs can be promoted through the path of *clinical verification–mechanism analysis–process optimization*. The overall effects of the compound prescription can be analyzed by metabolomics, and the potential synergistic or antagonistic effects can be predicted in combination with network pharmacology. The research on dose-effect relationship can be conducted, and the optimal dosing regimen can be determined in combination with pharmacokinetics. Ultimately, the leap from traditional experience to precision medicine can be achieved.

## 5. Conclusion

Breast hyperplasia is a common gynecological disease. In TCM theory, it is believed that women are based on blood, and blood stasis is the most common cause of gynecological diseases. *Qi* and blood are most closely related (*Qi* is the commander of blood and blood is the carrier of *Qi*), thus blood stasis can quickly lead to the stagnation of *Qi*. On the other hand, liver is a major blood storage organ d, and the obstruction of blood circulation immediately leads to liver system dysfunction. The stagnation of liver *Qi* exacerbates the stagnation of *Qi* in the entire body, which when it remains for a long time will affect other functions of the body, such as water metabolism, resulting in phlegm turbidness stagnation, and stagnation staying overtime transforms into heat and induces symptoms of redness, swelling, heat, and pain.

As a result, promoting blood circulation and resolving stasis are the most basic ways for treating breast hyperplasia, soothing the liver and regulating *Qi* are very important, clearing heat and detoxifying effectively eliminates the swelling and pain of breast hyperplasia. On the basis of the above treatments, other auxiliary treating methods are often combined, such as softening hardness, dispersing nodules, dispelling phlegm, discharging pus, reducing lumps and blood accumulation, etc.

*Angelica sinensis* has the effects of nourishing blood, promoting blood circulation, regulating menstruation and relieving pain. It is a commonly used gynecological herb and the most important herb for treating breast hyperplasia, and is mostly used in large doses.

According to the symptoms, the beginning of breast hyperplasia belongs to TCM pathological diseases, but in the early stages, it is often not taken seriously. As a result, most diagnosis and treatment are in the middle or late stages. The prolonged duration of the disease affects the body’s overall function, thus middle or late stage of MGH will show a deficiency state of *Qi*, blood, organs’ *Yin* and *Yang*, which is the pathological disease on a deficiency-based nature in TCM theory. Therefore, in the middle and late stages of breast hyperplasia, *Astragalus membranaceus* (solidifying skin surface, preventing excessive sweat, promoting diuresis and swelling, facilitating *Qi* circulation, eliminating toxins and pus, healing sore, accelerating muscle regeneration, nourishing *Qi*, blood, *Yin* and *Yang*) is also used in large doses, and the combination of *Angelica sinensis* and *Astragalus membranaceus* is commonly used in clinical practice.

High frequency herbs play an important role in breast hyperplasia treatment. The herbal pairs and core herbal combinations are fixed modes commonly used in breast hyperplasia treatment, and are the smallest prescription units that can have basic and excellent therapeutic effects of TCM prescriptions in treating breast hyperplasia.

Classical ancient prescriptions are derived from ancient medical works. After a long period of clinical verification, they have reliable efficacy and occupy an important position in the composition ratio of proprietary Chinese medicine. Classical and famous prescriptions derived from ancient medical books have acquired the advantages of modern pharmaceutical technology and process in terms of production and quality control, and have the advantages of standardized production, fixed quality and convenient application, which is an important direction for the modernization, industrialization and standardization of TCM. The core herbal combinations in this study are mostly derived from classic ancient prescriptions contained in ancient medical books, which provide important resources for the development of new compound drugs for breast hyperplasia.

## Acknowledgments

Thanks for the financial support provided by the National Natural Science Foundation, Hebei Natural Science Foundation, Scientific Research Project of Hebei Provincial Administration of Traditional Chinese Medicine.

## Author contributions

**Conceptualization:** Xujie Yang, Xiaohua Pei.

**Data curation:** Xujie Yang, Hong Zhang, Wanyue Zhang.

**Formal analysis:** Xujie Yang, Hong Zhang.

**Funding acquisition:** Xujie Yang.

**Investigation:** Xujie Yang.

**Methodology:** Xujie Yang, Xiaohua Pei.
